# Structural Elucidation and Cytotoxic Activity of New Monoterpenoid Indoles from *Gelsemium elegans*

**DOI:** 10.3390/molecules28062531

**Published:** 2023-03-10

**Authors:** Da Song, Jia-Jun Liang, Shi-Biao Pu, Pan-Pan Zhang, Yun-Lin Peng, Xia Liu, Ting-Ting Feng, Xiang Pu, Ying Zhou, Xiong-Wei Liu, Xin Wei

**Affiliations:** 1 School of Pharmacy, Guizhou University of Traditional Chinese Medicine, Guiyang 550025, China; 2 School of Humanities and Management, Guizhou University of Traditional Chinese Medicine, Guiyang 550025, China; 3 College of Chinese Materia Medica, Yunnan University of Chinese Medicine, Kunming 650500, China

**Keywords:** gelselegandines F and G, cytotoxicity, leukemic cells, *Gelsemium elegans*

## Abstract

Two new monoterpenoid indole alkaloids, gelselegandines F (**1**) and G (**2**), were isolated from the aerial parts of *Gelsemium elegans*. Their structures were elucidated by means of spectroscopic techniques and quantum chemical calculations. The ECD calculations were conducted at the B3LYP/6-311G(d,p) level and NMR calculations were carried out using the Gauge-Including Atomic Orbitals (GIAO) method. Structurally, the two new compounds possessed rare, cage-like, monoterpenoid indole skeletons. All isolated compounds and the total alkaloids extract were tested for cytotoxicity against four different tumor cell lines. The total alkaloids extract of *G. elegans* exhibited significant antitumor activity with IC_50_ values ranging from 32.63 to 82.24 ug/mL. In order to discover anticancer leads from the active extraction, both new indole compounds (**1–2**) were then screened for cytotoxicity. Interestingly, compound **2** showed moderate cytotoxicity against K562 leukemia cells with an IC_50_ value of 57.02 uM.

## 1. Introduction

Natural monoterpenoid indole alkaloids (MIAs) are widely distributed in many plants from the families Apocynaceae, Loganiaceae, and Rubiaceae [1,2,3]. Previous intensive studies of antitumor indole alkaloids have resulted in considerable groundbreaking discoveries in the past decades [4,5]. Especially due to star natural antitumor indoles entering the clinical forefront, such as vinblastine, vincristine, and vindesine, MIAs with remarkable biological activities and fascinating structures have attracted more and more attention from the pharmaceutical industry [6,7]. However, it is difficult to obtain clear NMR signals or fine single crystals from a large number of compounds, which limits the rapid determination of the stereoconfiguration of MIAs with multiple chiral centers. With the advancement of ECD and nuclear magnetic resonance (NMR) calculation methods, bioactive compound identification based on quantum chemical calculations has become the mainstream approach for the discovery of leading structures [8,9,10,11].

**Figure 1:**
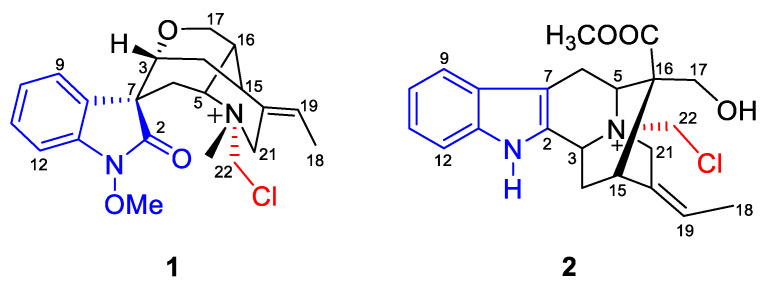
Structures of compounds **1–2**.

*Gelsemium elegans*, also known as “Gou Wen” or “Duan Chang Cao,” is a famous medicine in traditional Chinese medicine (TCM) [12]. *G. elegans* was historically used as a treatment for cancer, nervous pain, and skin ulcers by the folk people of China [12,13]. As part of our continuing research on MIAs [4,14,15], two new monoterpenoid indole alkaloids, gelselegandines F (**1**) and G (**2**), were isolated from the aerial parts of *G. elegans* (Figure 1). Both isolated compounds possessed the rare, cage-like, monoterpenoid indole skeleton. For the identification of their absolute configuration, spectroscopic techniques as well as ECD at the B3LYP/6-311G(d,p) level and NMR calculations using the Gauge-Including Atomic Orbitals (GIAO) method were carried out. In addition, the total alkaloids extract of *G. elegans* exhibited significant antitumor activity with IC_50_ values ranging from 32.63 to 82.24 ug/mL. Based on the antitumor effects of the total alkaloids extracts, new compounds **1** and **2** were then screened for cytotoxicity. Compound **2** showed moderate cytotoxicity against K562 leukemia cells with an IC_50_ value of 57.02 uM. This work provides a systematic approach to obtain an active compound from the total alkaloids of *G. elegans*, thereby supporting its traditional anti-cancer application.

## 2. Results and Discussion

### 2.1. Structure Elucidation

**Figure 2:**
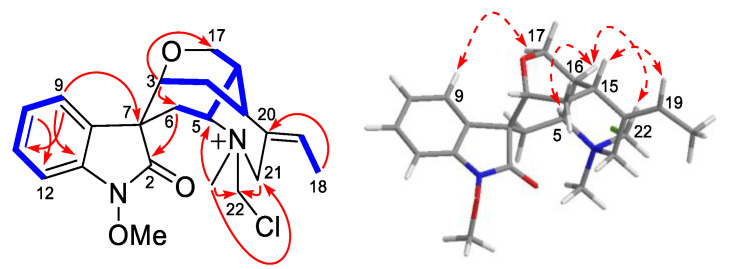
Selective and key HMBC (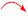), ^1^H-^1^H COSY (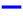) and ROESY (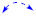) correlations of compound **1**.

Compound **1** displayed a positive reaction to Dragendorff’s reagent. Based on its HR-ESI-MS spectra, the quasi-molecular ion peak at *m/z* 403.1785 [M]^+^ (calcd for C_22_H_28_N_2_O_3_Cl^+^ 403.1783) assigned the molecular formula as C_22_H_28_N_2_O_3_Cl. The ^13^C and ^1^H NMR spectroscopic data of compound **1** (Table 1) displayed 22 carbon resonances for humantenine-type alkaloids, including three methyl groups (*δ*_C_ 13.4, 48.6, and 64.3), four methylenes (*δ*_C_ 27.8, 29.4, 65.6, and 54.7), nine methines (*δ*_C_ 33.1, 34.5, 71.0, 74.1, 108.7, 124.8, 126.8, 130.0, and 130.8), and five quaternary carbons (*δ*_C_ 57.0, 130.0, 131.2, 139.5, and 174.9). The above spectroscopic data were similar to those of the compound humantenine [16], except for the remaining CH_2_ (*δ*_C_ 69.4) and chlorine groups. Furthermore, the HMBC correlations (Figure 2) from *δ*_H_ 3.27 (*N*_4_ -CH_3_) and *δ*_H_ 4.40 (H-21) to *δ*_C_ 69.4 supported the linkage between the remaining -CH_2_Cl unit and *N*_4_.

The relative configuration of compound **1** was confirmed by NOE correlations. The *Z*-configured double bond of C-19/20 was established by the NOE correlation of *δ*_H_ 2.92 (H-15) with *δ*_H_ 6.03 (H-19) in its ROESY spectrum. In a molecular model, the rigid skeleton of a humantenine-type alkaloid required *β*-orientation for H-3 and H-15 to form the cage-like polycyclic system, which was consistent with the known analogue compound humantenine [16]. The NOE correlations of H-5/H-16/H-22 suggested the same orientation (*a*-orientation) of these protons. Meanwhile, the NOE correlation of H-9 with H-17 supported the relative configuration of C-7. Considering the solved relative configuration of compound **1,** its stereoconfiguration was assigned as two mutually enantiomers, 3*R*, 4*S*, 5*S*, 7*S*, 15*R*, 16*S* and 3S, 4*R*, 5*R*, 7*R*, 15*S*, 16*R*.

**Figure 3:**
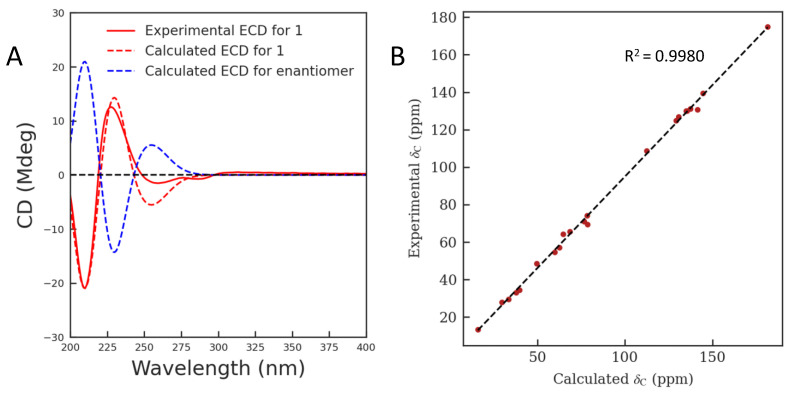
Quantum chemical calculations of compound **1**. (**A**) Calculated and experimental ECD spectra of compound **1**; (**B**) Regression analysis of experimental versus calculated ^13^C NMR chemical shifts of compound **1**.

The absolute configuration of compound **1** was finally solved by quantum chemical calculation (Supporting Information). As shown in Figure 3A, the calculated ECD spectrum for compound **1** (3*R*, 4*S*, 5*S*, 7*S*, 15*R*, 16*S*) matched well with the experimental spectrum. In addition, the ^13^C NMR calculation was further used to support its absolute configuration [17]. The results showed that the correlation coefficient (R^2^) from linear regression analysis between its calculated and experimental ^13^C NMR data was 0.9980 and the corrected mean absolute deviation (CMAD) was 1.55 (Figure 3B, Table S3). Based on the above findings, compound **1** was identified as shown in Figure 1 and named gelselegandine F.

**Figure 4:**
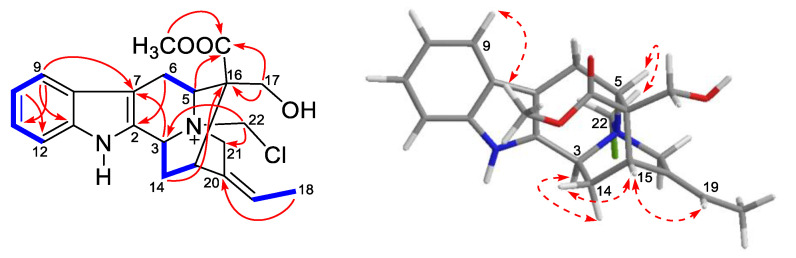
Selective and key HMBC (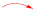), ^1^H-^1^H COSY (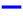) and ROESY (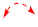) correlations of compound **2**.

The molecular formula of compound **2** was suggested as C_22_H_26_N_2_O_3_Cl by the quasi-molecular ion at *m/z* 401.1625 [M]^+^ (calcd for 401.1626). In the ^13^C and ^1^H NMR spectra of compound **2** (Table 1), a total of two methyl groups, five methylenes, eight methines, and seven quaternary carbon resonances were discovered. Such spectral data could be specifically assigned to sarpagine-type alkaloid *N*(b)-methylaknammidine [18], except the *N*_4_-methyl in *N*(b)-methylaknammidine was replaced by a CH_2_ (*δ*_C_ 67.8, C-22) moiety in compound **2**. Given the remaining chlorine group in the HR-ESI-MS analysis, the other end residue of C-22 was speculated to connect with chlorine. In addition, the HMBC correlations from *δ*_H_ 5.52 (H-22) to *δ*_C_ 61.6 (C-3) and *δ*_C_ 60.2 (C-21) further supported the previous reasoning and established the planar structure of compound **2** (Figure 4).

The relative configuration of compound **2** was confirmed by NOE correlations. In the ROESY spectrum of compound **2** (Figure 4), the NOE correlation of *δ*_H_ 5.73 (H-19) with *δ*_H_ 3.13 (H-15) suggested the *Z*-configured double bond of C-19/20. Meanwhile, NOE correlations of *δ*_H_ 3.07 (Ha-14) with *δ*_H_ 5.29 (H-3) and *δ*_H_ 2.53 (Hb-14) with *δ*_H_ 3.13 (H-15) established the *α*-orientations of H-3 and H-15, respectively. The relative configuration of quaternary C-16 was determined by the NOE correlation of H-9 with the hydrogen signal of a methoxy group. The NOE correlation between H-5 and H-22 supported the co-face of these protons (*a*-orientation). Thus, the relative configuration of compound **2** was identified and its absolute configuration was limited to two possibilities, 3*S*, 4*S*, 5*S*, 15*S*, 16*S* and 3*R*, 4*R*, 5*R*, 15*R*, 16*R*, which were mutual enantiomers.

**Figure 5:**
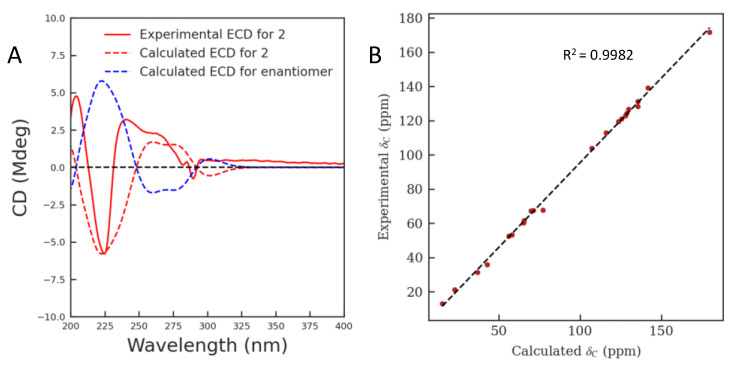
Quantum chemical calculations of compound **2**. (**A**) Calculated and experimental ECD spectra of compound **2**; (**B**) Regression analysis of experimental versus calculated ^13^C NMR chemical shifts of compound **2**.

Finally, the absolute configuration of compound **2** was identified by means of quantum chemical calculations [17] (Supporting Information). As shown in Figure 5A, the calculated ECD spectrum for compound **2** (3*S*, 4*S*, 5*S*, 15*S*, 16*S*) matched well with the experimental spectrum. In addition, the ^13^C NMR calculation showed that the correlation coefficient (R^2^) from linear regression analysis between its calculated and experimental ^13^C NMR data was 0.9982 and the corrected mean absolute deviation (CMAD) was 1.46 (Figure 5B, Table S3). Therefore, the structure of compound **2** was established and the new compound was named gelselegandine G.

### 2.2. The Cytotoxicity of Total Alkaloids and Compound 2

**Figure 6:**
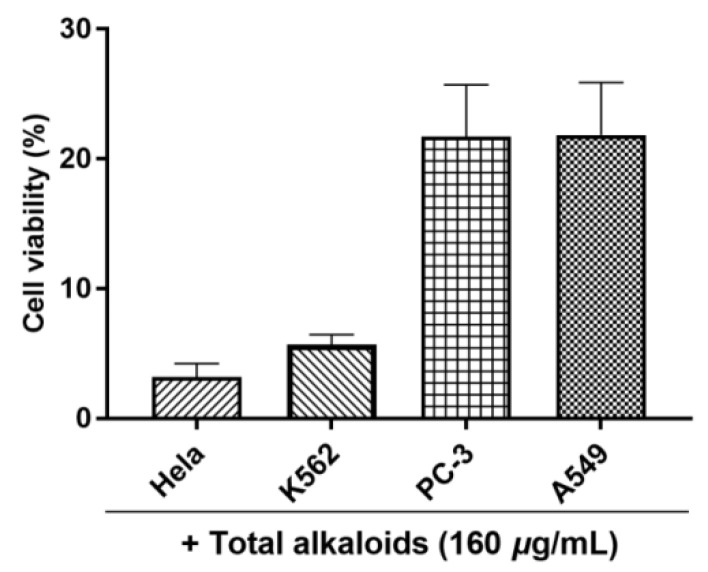
The cell viability of A549, Hela, K562, and PC-3 cells treated with the total alkaloids of *G. elegans* at a concentration of 160 ug/mL.

**Figure 7:**
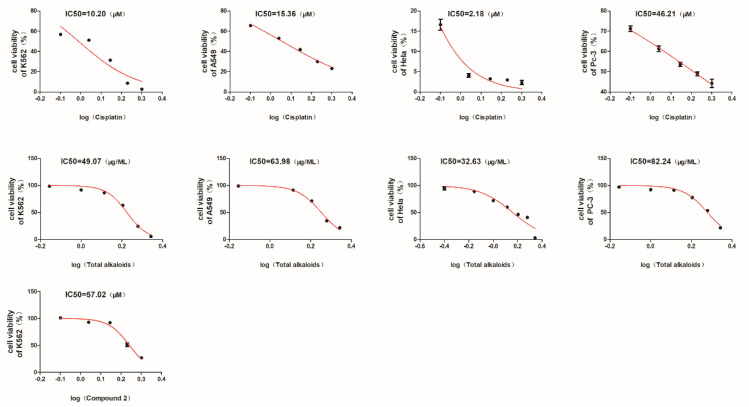
The IC_50_ values of the total alkaloids, compound **2,** and positive control drug cisplatin.

The inhibitory activity for the total alkaloids of *G. elegans* against four tumor cell lines (A549, Hela, K562, and PC-3) was preliminarily evaluated at a concentration of 160 ug/mL. Based on the results of MTT assay, the total alkaloids exhibited significant cytotoxicity towards the all Hela, K562, PC-3, and A549 cell lines (Figure 6). Under the treated concentration, the cell viability means of the four cell lines were lower than 30% (Figure 6). Especially for Hela cervical cancer cells and K562 leukemia cells, the total alkaloids of *G. elegans* showed the best inhibitory activity among of the four tested cell lines. Furthermore, the IC_50_ values of the total alkaloids were determined using MTT method. As shown in Figure 7, the IC_50_ values of the total alkaloids against K562, A549, Hela, and PC-3 cell lines were 49.07, 63.98, 32.63, and 82.24 ug/mL, respectively.

Based on the above antitumor clues of the total alkaloids, both new indole compounds **1** and **2** were then screened for cytotoxicity against A549, Hela, K562, and PC-3 cell lines. However, only compound **2** showed moderate cytotoxicity against K562 leukemia cells with an IC_50_ value of 57.02 uM (Figure 7), which was consistent with the better cytotoxicity of the total alkaloids toward Hela and K562 cells. Meaningful cytotoxicity against the other cell lines was not detected (IC_50_ > 100 uM). This study reports antitumor activity tracking from the total alkaloids extract to new monomer compounds, which might provide a new type of lead for the inhibition of K562 leukemia cells.

## 3. Experimental Section

### 3.1. General Experimental Procedures

Optical rotations were measured using an Autopol VI (serial #91058). IR spectra were determined using a Bruker Vertex 70 instrument with KBr pellets. HR-ESIMS data were obtained using a SHIMADZU UPLC-IT-TOF. UV spectra were measured using a SHIMADZU UV-2401PC. The 1D and 2D NMR spectra were measured on a Bruker Avance NEO (400 MHz). TLC analyses were carried out on precoated silica gel GF-254 plates and column chromatography was performed on 200–300 mesh silica gel (Qingdao Marine Chemical Plant, Qingdao, China), MCI-gel (Mitsubishi Chemical Co., Ltd., Tokyo, Japan), and ODS-gel (50 µm, YMC, Kyoto, Japan). HPLC was carried out SEP LC-52 using an MWD UV detector (Separation Technology Co., Ltd., Beijing, China) and semi-preparative C18 columns (250 × 10 mm).

### 3.2. Plant Material

The aerial parts of *G. elegans* were purchased from Kunming Zhifen Biotechnology Co., Ltd. (Kunming, China) in April 2021, and identified by An-Rui Lou from Kunming Zhifen Biotechnology Co., Ltd. A voucher specimen (No. WX_20210401) was deposited in Guizhou University of Traditional Chinese Medicine.

### 3.3. Extraction and Isolation

The aerial parts of *G. elegans* (20 kg) were extracted with 95% ethanol (30 L × 3) under reflux conditions at 70 °C, for two hours each time. After removal of the organic solvent under reduced pressure, the crude extract (3893 g) was obtained. The ethanol extract was dissolved in H_2_O and acidified with dilute acid water to pH 2, then basified with NH_3_.H_2_O to pH 10 and extracted with CH_2_Cl_2_ to obtain the total alkaloids (221 g). The total alkaloids were separated by flash silica gel column chromatography using a MeOH/CH_2_Cl_2_ (1:100–1:0, *v:v*) gradient to give 25 fractions (L1–L25). Then, L-9 and L-10 were merged together and separated by ODS column chromatography using a MeOH/H_2_O (2:3–1:0, *v:v*) gradient to give 15 sub-fractions. Sub-fraction 15 was successively separated by MCI column chromatography using a MeOH/H_2_O (1:1–1:0, *v:v*) gradient and by HPLC preparative chromatographic isocratic elution with MeOH/H_2_O (3:7, *v:v*) to give compounds **1** (20.0 mg) and **2** (47.0 mg).

#### 3.3.1. Gelselegandine F (1)

Amorphous solid; [α]^18^_D_ -102.1 (*c* 0.06, MeOH); UV (MeOH) *λ*_max_ 207, 254, 279 nm; IR (KBr) *ν*_max_ 3432, 2926, 1718, 1619, 1384, and 1077 cm^−1^; HRESIMS *m/z* 403.1785 [M]^+^ (calcd for C_22_H_28_N_2_O_3_Cl^+^ 403.1783); ^1^H and ^13^C NMR data, see Table 1.

#### 3.3.2. Gelselegandine G (2)

Amorphous solid; [α]^18^_D_ -18.1 (*c* 0.07, MeOH); UV (MeOH) *λ*_max_ 219, 269, 278, 285 nm; IR (KBr) *ν*_max_ 3430, 2926, 1729, 1623, 1457, 1234, and 1074 cm^−1^; HRESIMS *m/z* 401.1625 [M]^+^ (calcd for C_22_H_26_N_2_O_3_Cl^+^ 401.1626); ^1^H and ^13^C NMR data, see Table 1.

### 3.4. ECD Calculation

The ECD calculations of compounds **1** and **2** were carried out using Gaussian 09 (Supporting Information). At first, all conformers were optimized at PM6. Room temperature equilibrium populations were calculated according to the Boltzmann distribution law (Supporting Information), based on which dominant conformers of the population (over 1%) were kept. The chosen conformers were further optimized at B3LYP/6-31G(d,p) in gas phase. Vibrational frequency analysis confirmed the stable structures. ECD calculations were conducted at the B3LYP/6-311G(d,p) level in methanol with the IEFPCM model using time-dependent density functional theory (TD-DFT). The ECD spectrum was simulated using the ECD/UV analysis tool by overlapping Gaussian functions for each transition (Supporting Information). The spectra of the enantiomers were produced directly by mirror inversion about the horizontal axis [17].

### 3.5. ^13^C NMR Calculation

The conformers of compounds **1** and **2** were directly derived from the previous ECD calculations. NMR calculations were carried out using the Gauge-Including Atomic Orbitals (GIAO) method at the mPW1PW91/6-311+G(2d,p) level in methanol simulated by the IEFPCM model (Supporting Information). The TMS-corrected NMR chemical shift values were averaged according to Boltzmann distribution and fitted to the experimental values by linear regression. The calculated ^13^C-NMR chemical shift value of TMS in methanol was 187.37 ppm [17].

### 3.6. Cell Lines and Cell Culture

All cell lines were purchased from the Chinese Academy of Sciences, Kunming Cell Bank. All cells were cultured in RPMI-1640 or DMEM media (Gibco, Beijing, China) supplemented with 10% fetal bovine serum, 1% glutamine, 100 U/mL penicillin, and 100 mg/mL streptomycin in a humidified atmosphere with 5% CO_2_ at 37 °C. The compounds were stored at −20 °C after being dissolved in DMSO. Cisplatin was purchased from Aladdin Company (Shanghai, China).

### 3.7. Cell Viability Assay

Cells in the logarithmic growth phase were washed with PBS and the cell density was regulated as 5 × 10^3^ cells/mL. The cells (80 μL/well) were inoculated in 96-well plates and incubated at 37 °C under 5% CO_2_. The cells were cultured with total alkaloids (1.25, 2.5, 5, 10, 20, 40, 80, 160, 320, and 640 μg/mL), compounds **1** and **2**, and positive control cisplatin (100, 50, 25, 12.5, and 6.25 μM, respectively, for monomer compounds) in medium for 48 h. Each group had equal five wells and five blank wells without cells were set at the same time. Then, 10 *μ*L of MTT (Solarbio, Beijing Solarbio Science & Technology Co., Ltd., Beijing, China) was added to each well and the cells were incubated for 4 h at 37 °C under 5% CO_2_. The absorbance of each well was measured at 490 nm using an automatic microplate reader (Thermo Fisher Scientific 3020, Waltham, MA, USA). Cell viability % = (OD value of detection well − background OD value)/(OD value of blank control group − background OD value) × 100%.

## 4. Conclusions

In conclusion, as part of our continuing study on MIAs from traditional Chinese medicine, two new monoterpenoid indole alkaloids, gelselegandines F (**1**) and G (**2**), were isolated from the aerial parts of *G. elegans*. For their absolute configuration identification, ECD and NMR calculations were used together with spectroscopic techniques. The cell viability assay results exhibited significant and broad antitumor effects of the total alkaloids from *G. elegans*, and the IC_50_ values of the total alkaloids against K562, A549, Hela, and PC-3 cell lines were 49.07, 63.98, 32.63, and 82.24 ug/mL, respectively. Based on the antitumor clues provided by the total alkaloids, both new indole compounds **1** and **2** were then screened for cytotoxicity against the above four cell lines. Compound **2** showed moderate cytotoxicity against K562 leukemia cells with an IC_50_ value of 57.02 uM. This work reports cytotoxic activity tracking from a total alkaloids extract to new monomer compounds, thus shedding light on the antitumor ingredients of *G. elegans*.

## Figures and Tables

**Table 1:** ^1^ H (400 MHz) and ^13^ C NMR (100 MHz) data for compounds **1–2** (*δ* in ppm, *J* in Hz).

NO.	Compound 1 ^a^		Compound 2 ^a^	
	*δ*_H_	*δ*_C_	*δ*_H_	*δ*_C_
2		174.9		131.2
3	3.65 (d, *J* = 7.0, 1H)	74.1	5.29 (d, *J* = 10.3, 1H)	61.6
5	4.56 (m, 1H)	71.0	3.93 (m, 1H)	67.4
6	2.78 (dd, *J* = 17.3, 4.3, 1H) 2.64 (dd, *J* = 17.3, 8.2, 1H)	29.4	3.26 (dd, *J* = 18.3, 4.8, 1H) 3.91 (d, *J* = 4.6, 1H)	21.1
7		57.0		103.9
8		131.2		126.7
9	7.54 (d, *J* = 7.5, 1H)	126.8	7.51 (d, *J* = 7.9, 1H)	119.5
10	7.20 (td, *J* = 7.6, 0.9, 1H)	124.8	7.11 (m, 1H)	121.2
11	7.41 (td, *J* = 7.7, 0.9, 1H)	130.0	7.21 (m, 1H)	124.3
12	7.11 (d, *J* = 7.7, 1H)	108.7	7.40 (d, *J* = 8.2, 1H)	112.9
13		139.5		139.1
14	2.41 (ddd, *J* = 15.7, 11.5, 7.3, 1H) 2.28 (dd, *J* = 15.7, 5.5, 1H)	27.8	2.53 (m, 1H) 3.07 (dd, *J* = 13.3, 4.4, 1H)	31.3
15	2.92 (m, 1H)	34.5	3.13 (d, *J* = 4.1, 1H)	35.9
16	2.86 (dd, *J* = 10.1, 5.1, 1H)	33.1		53.3
17	4.33 (d, *J* = 11.5, 1H) 4.19 (dd, *J* = 11.5, 4.8, 1H)	65.6	3.85 (m, 2H)	67.5
18	1.86 (d, *J* = 6.9, 3H)	13.4	1.73 (d, *J* = 7.0, 3H)	13.0
19	6.03 (q, *J* = 6.9, 1H)	130.8	5.73 (qd, *J* = 7.0,4.5, 1H)	122.9
20		130.0		128.4
21	5.48 (d, *J* = 11.1, 1H) 4.40 (d, *J* = 14.5, 1H)	54.7	4.77 (d, *J* = 16.4, 1H), 4.60 (d, *J* = 16.2, 1H)	60.2
22	5.46 (s, 2H)	69.4	5.52 (d, *J* = 10.4, 1H) 5.40 (d, *J* = 10.4, 1H)	67.8
*N*_1_-OCH_3_	4.03 (s, 3H)	64.3		
*N*_4_-CH_3_	3.27 (s, 3H)	48.6		
COOCH_3_			3.01 (s, 3H)	52.7
COOCH_3_				171.7

^a^ Recorded in CD_3_OD.

## Data Availability

The research data were available in Supporting Information.

## Supplementary Materials

The following supporting information can be downloaded at: https://www.mdpi.com/article/10.3390/molecules28062531/s1. Parts of calculations and experiments, 1D and 2D NMR spectra, HRESIMS, and UV and ECD spectra of compounds **1** and **2** are available as Supporting Information. The Figures S1–S27 and Tables S1–S6 were listed in Supporting Information [19,20].

## Sample Availability

Samples of the compounds are not available from the authors.
