# Supplementary material for: Structural Elucidation and Cytotoxic Activity of New Monoterpenoid Indoles from Gelsemium elegans
Source: Molecules. 2023 Mar 10;28(6):2531. doi: 10.3390/molecules28062531 (PMC10055825; doi:10.3390/molecules28062531)
Supplement: Supplementary file 1 [file molecules-28-02531-s001.zip › molecules-2229947-supplementary.pdf]

Structural elucidation of new monoterpenoid indoles from *Gelsemium elegans* using quantum chemical calculations and their antitumor activities

Da Song,[a,b,1] Jia-Jun Liang,[a,1] Shi-Biao Pu,[c,1] Pan-Pan Zhang,[a] Yun-Lin Peng,[a] Xia Liu,[a] Ting-Ting Feng,[a] Xiang Pu,[a] Ying Zhou,[a] Xiong-Wei Liu[a,*] and Xin Wei [a,*]

[a]*School of Pharmacy, Guizhou University of Traditional Chinese Medicine, Guiyang 550025, People's Republic of China.*

[b]*School of Humanities and Management, Guizhou University of Traditional Chinese Medicine, Guiyang 550025, People's Republic of China.*

[c]*College of Chinese Materia Medica, Yunnan University of Chinese Medicine, Kunming, 650500, People's Republic of China.*

---

*Corresponding author: ashevy0819@163.com (X.-W. Liu); sfweixin@163.com (X. Wei).

[1]These authors contributed equally to this work.

Table of contents

1. Experimental and computed ^{13}C-NMR chemical shifts 1

2. ECD calculations of 1 and 2 3

2.1. Computational methods 3
2.2. Energies and coordinates 5
2.3. Experimental and calculated ECD spectra of 1 and 2 13

3. Supplementary figures 13

Figure S6. ^{1} H NMR spectrum of 1 (CD_3OD, 400 MHz) 14
Figure S7. ^{13}C NMR spectrum of 1 (CD_3OD, 100 MHz) 15
Figure S8. HSQC spectrum of 1 (CD_3OD, 400 MHz). 16
Figure S9. HMBC spectrum of 1 (CD_3OD, 400 MHz). 17
Figure S10. ROESY spectrum of 1 (CD_3OD, 400 MHz). 18
Figure S11. ^{1}H-^{1}H COSY spectrum of 1 (CD_3OD, 400 MHz). 19
Figure S12. HR-ESI-MS spectrum of 1 20
Figure S13. ORD spectrum of 1 21
Figure S14. UV spectrum of 1 22
Figure S15. IR spectrum of 1 23
Figure S16. ECD spectrum of 1 24
Figure S17. ^{1}H NMR spectrum of 2 (CD_3OD, 400 MHz) 25
Figure S18. ^{13}C NMR spectrum of 2 (CD_3OD, 100 MHz) 26
Figure S19. HSQC spectrum of 2 (CD_3OD, 400 MHz). 27
Figure S20. HMBC spectrum of 2 (CD_3OD, 400 MHz). 28
Figure S21. ROESY spectrum of 2 (CD_3OD, 400 MHz). 29
Figure S22. ^{1}H-^{1}H spectrum of 2 (CD_3OD, 400 MHz). 30
Figure S23. HR-ESI-MS spectrum of 2 31
Figure S24. ORD spectrum of 2 32
Figure S25. UV spectrum of 2 33
Figure S26. IR spectrum of 2 34
Figure S27. ECD spectrum of 2 35
References 35

1. Experimental and computed ^{13}C-NMR chemical shifts

The TMS-corrected computed ^{13}C-NMR chemical shifts of compounds **1** and **2** were fitted to the experimental values by Ordinary Least Squares (OLS) Linear Regression method in order to remove systematic error that results from the conformational search and random error from experimental conditions (**Tables S1-S3**).

Table S1 Experimental and computed ^{13}C-NMR chemical shifts of **1**.

Position	Experimental	calculated
2	174.9	174.7056359
3	74.1	73.77670849
5	71.0	72.20161502
6	29.4	30.0031432
7	57.0	58.48536649
8	131.2	131.5162235
9	126.8	124.9741055
10	124.8	123.6012893
11	130.0	129.2364188
12	108.7	107.2296033
13	139.5	138.4664691
14	27.8	26.33057925
15	34.5	36.15480094
16	33.1	34.37886398
17	65.6	64.24414924
18	13.4	12.94679213
19	130.8	135.4254288
20	130.0	129.5051204
21	54.7	55.79416295
22	69.4	74.20490931
N_2-OMe	64.3	60.59474299
N_4-Me	48.6	45.82387137

Table S2 Experimental and computed ^{13}C-NMR chemical shifts of **2**.

Position	Experimental	calculated
2	131.2	130.5271157
3	61.6	61.3739485
5	67.4	65.54997428
6	21.1	18.97712099
7	103.9	102.6619612
8	126.7	125.032549
9	119.5	118.7670812
10	121.2	120.6764227
11	124.3	123.8913627

12	112.9	111.3440135
13	139.1	136.7802114
14	31.3	32.93263895
15	35.9	38.85662307
16	53.3	54.0289765
17	67.5	66.92161865
18	13.0	11.5087365
19	122.9	123.0622
20	128.4	130.5439096
21	60.2	61.01637075
22	67.8	72.73629931
COOMe	171.7	174.3550486
COOMe	52.7	52.05581688

Relatively higher R^2 and lower CMAD and CLAD values were shown in both ^{13}C-NMR Ordinary Least Squares Linear Regression (OLS-LR) for **1**, which indicated that this configuration was the correct structure.

Table S3 Statistics of Ordinary Least Squares Linear Regression (OLS-LR) of experimental and computed ^{13}C-NMR chemical shifts of **1** and **2**.

Type	Compound	CMAD[a]	CLAD[b]	R^2	*RMSD*	*F*	*p* value	*Slope*
CNMR	**1**	1.55	4.80	0.9980	2.1069	9962.11	< 0.01	0.9767
CNMR	**2**	1.46	4.94	0.9982	1.9124	11277.73	< 0.01	0.9898

[a] CMAD = corrected mean absolute deviation, computed as $(1/n)\sum_i^n |\delta_{calc} - \delta_{exp}|$, where δ_{calc} and δ_{exp} refer to the calculated and experimental chemical shifts. [b] CLAD = corrected largest absolute deviation, computed as $\max(|\delta_{calc} - \delta_{exp}|)$.

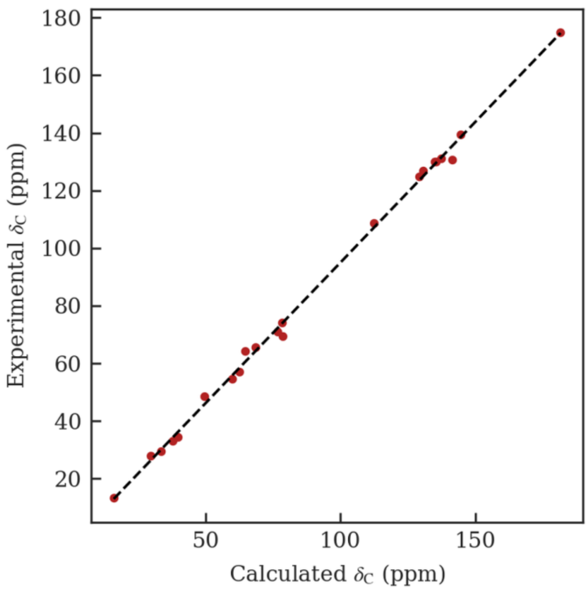

Figure S1 Regression analysis of experimental versus calculated ^{13}C- NMR chemical shifts of **1**.

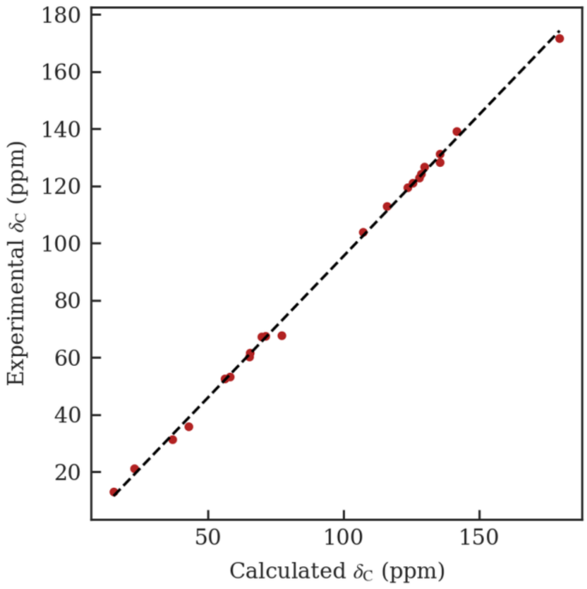

Figure S2 Regression analysis of experimental versus calculated ^{13}C- NMR chemical shifts of **2**.

2. ECD calculations of 1 and 2

2.1. Computational methods

2.1.1. Conformational analysis

Conformational analysis for **1** and **2** (**Figure S2**) were performed using systematic algorithm by

Confab [1] at MMFF94 force field with RMSD threshold of 0.2 Å and energy window of 7 kcal/mol.

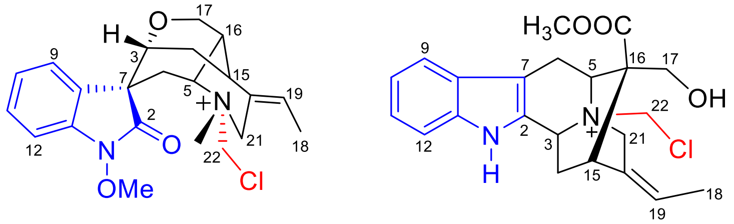

1 **2**

Figure S3 Chemical structure of compounds **1** and **2**.

2.1.2. ECD calculation

The theoretical calculations were carried out using Gaussian 09[2]. At first, all conformers were optimized at PM6, and the net charge was set as one. Room-temperature equilibrium populations were calculated according to Boltzmann distribution law (eq.1), based on which dominative conformers of population over 1% were kept. The chosen conformers were further optimized at B3LYP/6-31G(d,p) in gas phase. Vibrational frequency analysis confirmed the stable structures. ECD calculations were conducted at B3LYP/6-311G(d,p) level in methanol with IEFPCM model using Time-dependent Density functional theory (TD-DFT). Rotatory strengths for 30 excited states were calculated (**Tables S4** and **S5**). The ECD spectrum was simulated using the ECD/UV analysis tool by overlapping Gaussian functions for each transition according to (eq.2).

$$\frac{N_i}{N} = \frac{g_i e^{-\frac{E_i}{k_B T}}}{\sum g_i e^{-\frac{E_i}{k_B T}}} \qquad \textbf{(eq.3)}$$

where N_i is the number of conformer i with energy E_i and degeneracy g_i at temperature T, and k_B is Boltzmann constant.

$$\Delta\varepsilon(E) = \frac{1}{2.297\times10^{-39}} \times \frac{1}{\sqrt{2\pi\sigma}} \sum_{i}^{A} \Delta E_i R_i e^{-\left(\frac{E-E_i}{2\sigma}\right)^2} \qquad \textbf{(eq.4)}$$

where σ represents the width of the band at 1/e height, while ΔE_i and R_i are the excitation energies and rotatory strengths for transition i, respectively. The σ and UV-shift values were set 0.27 eV and -22 nm, respectively. The spectrum of the enantiomers were produced directly by mirror inversion about the horizontal axis.

2.1.3. NMR calculation

The structures were directly derived from the previous ECD calculations. NMR calculations were carried out using the Gauge-Including Atomic Orbitals (GIAO) method at mPW1PW91/6-311+G(2d,p) level in Methanol simulated by the IEFPCM model. The TMS-corrected NMR chemical shift values were averaged according to Boltzmann distribution and fitted to the experimental values by linear regression. The calculated ^{13}C-NMR chemical shift values of TMS in Methanol were 187.37 ppm, respectively.

2.2. Energies and coordinates

2.2.1. Energies at B3LYP theory level

Structures for ECD calculation were shown in **Tables S4** and **S5**.

Table S4 Energies of configuration **1** at B3LYP/6-311G(d,p) in methanol.

Conformer	Structure	E (Hartree)	E (kcal/mol)	Population (%)
**1a**	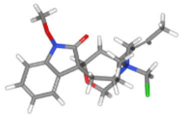	-1650.644342	-1035794.954	54.35
**1b**	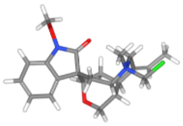	-1650.643342	-1035794.327	18.85
**1c**	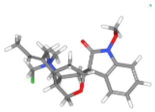	-1650.643674	-1035794.536	26.81

Table S5 Energies of configuration **2** at B3LYP/6-311G(d,p) in methanol.

Conformer	Structure	E (Hartree)	E (kcal/mol)	Population (%)
**2a**	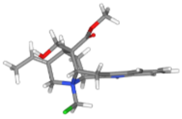	-1649.512692	-1035084.833	28.99
**2b**	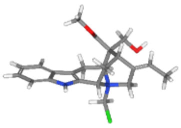	-1649.512243	-1035084.552	18.02
**2c**	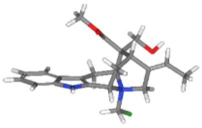	-1649.512894	-1035084.96	35.9
**2d**	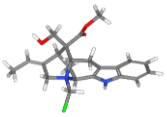	-1649.512192	-1035084.52	17.08

2.2.2. Coordinates at B3LYP theory level

Table S6 Standard orientations of configuration **1** for ECD calculation.

Conformer **1a**

Center Number	Atomic Number	Atomic Type	Coordinates (Angstroms) X	Y	Z
1	6	0	4.451669	-2.373832	0.330859
2	6	0	5.316991	-1.412926	-0.197402
3	6	0	4.853633	-0.140931	-0.555999
4	6	0	3.503550	0.106258	-0.368137
5	6	0	2.609471	-0.841573	0.139847
6	6	0	3.089253	-2.092906	0.507407
7	7	0	2.783730	1.291802	-0.627084
8	6	0	1.439695	1.157830	-0.433084
9	6	0	1.207792	-0.230694	0.220755
10	6	0	0.788313	-0.130571	1.735261
11	6	0	0.214214	-1.088676	-0.612740
12	6	0	-0.370776	0.815914	2.090676
13	6	0	-1.785339	0.197644	1.867435
14	6	0	-2.716802	1.099730	1.088748
15	6	0	-2.314421	1.262662	-0.344401
16	7	0	-2.219029	-0.098774	-1.078496
17	6	0	-1.306318	-1.132528	-0.318412
18	6	0	-1.677540	-1.184332	1.184891
19	6	0	-3.761960	1.723511	1.656886
20	6	0	-4.757834	2.661994	1.041855
21	6	0	-0.695047	-2.056175	1.981605
22	8	0	0.569805	-1.444477	2.241872
23	8	0	0.598284	1.997285	-0.750922
24	6	0	-3.640584	-0.624332	-1.157135
25	17	0	-3.811155	-2.156344	-2.050009
26	6	0	-1.716495	0.177126	-2.473445
27	8	0	3.318561	2.304292	-1.395055
28	6	0	3.528131	3.499137	-0.610841
29	1	0	4.836565	-3.349769	0.609440
30	1	0	6.367764	-1.650973	-0.332092
31	1	0	5.511185	0.614194	-0.971706
32	1	0	2.427783	-2.839088	0.935305
33	1	0	1.676972	0.216261	2.269198
34	1	0	0.401579	-0.936287	-1.678040
35	1	0	0.512571	-2.126575	-0.437871
36	1	0	-0.256103	1.756496	1.546377
37	1	0	-0.269980	1.060830	3.152243
38	1	0	-2.235711	0.011035	2.848136
39	1	0	-3.007458	1.868050	-0.930321
40	1	0	-1.310272	1.686798	-0.439655
41	1	0	-1.631582	-2.071598	-0.770372
42	1	0	-2.651242	-1.682630	1.271120
43	1	0	-3.904261	1.555725	2.725272
44	1	0	-4.642400	2.794589	-0.037580
45	1	0	-5.780003	2.313605	1.235227
46	1	0	-4.677597	3.653401	1.505665
47	1	0	-1.159000	-2.284564	2.949439
48	1	0	-0.517624	-3.014264	1.474370
49	1	0	-4.231594	0.131234	-1.672763
50	1	0	-4.011743	-0.760135	-0.145255
51	1	0	-2.478442	0.756130	-2.998846
52	1	0	-0.801337	0.761890	-2.400989
53	1	0	-1.548904	-0.763874	-2.996439
54	1	0	4.003235	4.195610	-1.303385
55	1	0	2.571804	3.903017	-0.268090
56	1	0	4.191761	3.296768	0.235886

Rotational constants (GHZ): 0.2764617 0.1198577 0.110863

Leave Link 202 at Wed Dec 21 11:49:19 2022, MaxMem= 6442450944 cpu:

(Enter /public1/home/sc81486/software/g09/l301.exe)

Standard basis: 6-311G(2d,p) (5D, 7F)

[illegible]

Conformer **1b**

Center Number	Atomic Number	Atomic Type	Coordinates (Angstroms) X	Y	Z
1	6	0	4.757243	-1.948216	-0.813706
2	6	0	5.512484	-0.773252	-0.799529
3	6	0	4.920006	0.464106	-0.518548
4	6	0	3.557640	0.460283	-0.267384
5	6	0	2.771706	-0.696583	-0.293356
6	6	0	3.379104	-1.918555	-0.556159
7	7	0	2.722036	1.550640	0.052577
8	6	0	1.401403	1.212874	0.122320
9	6	0	1.320385	-0.335449	0.035247
10	6	0	0.930395	-1.001703	1.406885
11	6	0	0.394332	-0.791462	-1.126790
12	6	0	-0.308982	-0.453749	2.134932
13	6	0	-1.659856	-1.032646	1.612456
14	6	0	-2.702463	0.026533	1.330796
15	6	0	-2.362562	0.902144	0.164862
16	7	0	-2.139575	0.081530	-1.126035
17	6	0	-1.110077	-1.107859	-0.926182
18	6	0	-1.431798	-1.909472	0.361685
19	6	0	-3.791327	0.184229	2.101960
20	6	0	-4.895041	1.193926	1.996115
21	6	0	-0.345256	-2.950517	0.673075
22	8	0	0.855502	-2.413924	1.228378
23	8	0	0.475806	2.016373	0.217872
24	6	0	-3.436394	-0.581629	-1.560081
25	17	0	-4.759236	0.555225	-1.921691
26	6	0	-1.668414	1.025849	-2.202004
27	8	0	3.138017	2.852372	-0.131161
28	6	0	3.243447	3.550257	1.129301
29	1	0	5.240974	-2.896541	-1.025484
30	1	0	6.577345	-0.815346	-1.007398
31	1	0	5.490880	1.385748	-0.508095
32	1	0	2.804533	-2.839085	-0.551240
33	1	0	1.793104	-0.861731	2.063509
34	1	0	0.547039	-0.144945	-1.993594
35	1	0	0.788621	-1.760871	-1.446374
36	1	0	-0.302316	0.638311	2.096737
37	1	0	-0.206823	-0.725753	3.189733
38	1	0	-2.059386	-1.707803	2.376642
39	1	0	-3.138088	1.626647	-0.077162
40	1	0	-1.416377	1.429100	0.311311
41	1	0	-1.355111	-1.743674	-1.783067
42	1	0	-2.347461	-2.485298	0.182036
43	1	0	-3.883567	-0.493084	2.952349
44	1	0	-4.826194	1.842168	1.119214
45	1	0	-5.869267	0.690431	1.970161
46	1	0	-4.902193	1.834464	2.887463
47	1	0	-0.761441	-3.657920	1.401564
48	1	0	-0.083827	-3.529423	-0.223758
49	1	0	-3.771782	-1.221927	-0.749395
50	1	0	-3.234245	-1.153891	-2.464425
51	1	0	-2.468545	1.733966	-2.412294
52	1	0	-0.796318	1.560377	-1.831354
53	1	0	-1.432735	0.457366	-3.103926
54	1	0	3.631700	4.533783	0.860386
55	1	0	2.258929	3.648966	1.594120
56	1	0	3.941878	3.038737	1.799216

```
Rotational constants (GHZ):      0.3088845      0.1136173      0.10589
Leave Link  202 at Wed Dec 21 11:42:55 2022, MaxMem=  6442450944 cpu:
(Enter /public1/home/sc81486/software/g09/l301.exe)
```

Conformer **1c**

Center Number	Atomic Number	Atomic Type	Coordinates (Angstroms) X	Y	Z
1	6	0	4.757243	-1.948216	-0.813706
2	6	0	5.512484	-0.773252	-0.799529
3	6	0	4.920006	0.464106	-0.518548
4	6	0	3.557640	0.460283	-0.267384
5	6	0	2.771706	-0.696583	-0.293356
6	6	0	3.379104	-1.918555	-0.556159
7	7	0	2.722036	1.550640	0.052577
8	6	0	1.401403	1.212874	0.122320
9	6	0	1.320385	-0.335449	0.035247
10	6	0	0.930395	-1.001703	1.406885
11	6	0	0.394332	-0.791462	-1.126790
12	6	0	-0.308982	-0.453749	2.134932
13	6	0	-1.659856	-1.032646	1.612456
14	6	0	-2.702463	0.026533	1.330796
15	6	0	-2.362562	0.902144	0.164862
16	7	0	-2.139575	0.081530	-1.126035
17	6	0	-1.110077	-1.107859	-0.926182
18	6	0	-1.431798	-1.909472	0.361685
19	6	0	-3.791327	0.184229	2.101960
20	6	0	-4.895041	1.193926	1.996115
21	6	0	-0.345256	-2.950517	0.673075
22	8	0	0.855502	-2.413924	1.228378
23	8	0	0.475806	2.016373	0.217872
24	6	0	-3.436394	-0.581629	-1.560081
25	17	0	-4.759236	0.555225	-1.921691
26	6	0	-1.668414	1.025849	-2.202004
27	8	0	3.138017	2.852372	-0.131161
28	6	0	3.243447	3.550257	1.129301
29	1	0	5.240974	-2.896541	-1.025484
30	1	0	6.577345	-0.815346	-1.007398
31	1	0	5.490880	1.385748	-0.508095
32	1	0	2.804533	-2.839085	-0.551240
33	1	0	1.793104	-0.861731	2.063509
34	1	0	0.547039	-0.144945	-1.993594
35	1	0	0.788621	-1.760871	-1.446374
36	1	0	-0.302316	0.638311	2.096737
37	1	0	-0.206823	-0.725753	3.189733
38	1	0	-2.059386	-1.707803	2.376642
39	1	0	-3.138088	1.626647	-0.077162
40	1	0	-1.416377	1.429100	0.311311
41	1	0	-1.355111	-1.743674	-1.783067
42	1	0	-2.347461	-2.485298	0.182036
43	1	0	-3.883567	-0.493084	2.952349
44	1	0	-4.826194	1.842168	1.119214
45	1	0	-5.869267	0.690431	1.970161
46	1	0	-4.902193	1.834464	2.887463
47	1	0	-0.761441	-3.657920	1.401564
48	1	0	-0.083827	-3.529423	-0.223758
49	1	0	-3.771782	-1.221927	-0.749395
50	1	0	-3.234245	-1.153891	-2.464425
51	1	0	-2.468545	1.733966	-2.412294
52	1	0	-0.796318	1.560377	-1.831354
53	1	0	-1.432735	0.457366	-3.103926
54	1	0	3.631700	4.533783	0.860386
55	1	0	2.258929	3.648966	1.594120
56	1	0	3.941878	3.038737	1.799216

Rotational constants (GHZ): 0.3088845 0.1136173 0.105895
Leave Link 202 at Wed Dec 21 11:42:55 2022, MaxMem= 6442450944 cpu:
(Enter /public1/home/sc81486/software/g09/l301.exe)

Table S7 Standard orientations of configuration **2** for ECD calculation.

Conformer **2a**

Center Number	Atomic Number	Atomic Type	Coordinates (Angstroms) X	Y	Z
1	6	0	-5.376860	-0.347451	-0.852848
2	6	0	-5.730619	-0.443549	0.509582
3	6	0	-4.766759	-0.570354	1.502084
4	6	0	-3.429636	-0.598675	1.094244
5	6	0	-3.050250	-0.504528	-0.272003
6	6	0	-4.048912	-0.377792	-1.253913
7	7	0	-2.269550	-0.719655	1.847032
8	6	0	-1.186266	-0.706620	0.990867
9	6	0	-1.611935	-0.568514	-0.306997
10	6	0	0.238806	-0.714619	1.449472
11	7	0	1.108640	-1.196160	0.258876
12	6	0	0.786213	-0.289859	-0.973806
13	6	0	-0.646834	-0.564926	-1.452984
14	6	0	0.738243	0.692568	1.887376
15	6	0	2.952050	0.328237	0.971482
16	6	0	2.595295	-1.094688	0.613697
17	6	0	4.156360	0.700494	1.411236
18	6	0	1.754330	1.233544	0.856358
19	6	0	5.357783	-0.179058	1.596778
20	6	0	1.175022	1.199442	-0.595255
21	6	0	2.223268	1.693601	-1.643847
22	8	0	3.224223	0.704620	-1.833227
23	6	0	0.781049	-2.655299	0.011477
24	17	0	1.630163	-3.355316	-1.388379
25	6	0	0.003244	2.199323	-0.791833
26	8	0	-0.574663	2.334756	-1.849577
27	8	0	-0.232314	2.967518	0.279664
28	6	0	-1.260467	3.976621	0.111518
29	1	0	-6.160558	-0.249008	-1.597690
30	1	0	-6.779438	-0.418189	0.789379
31	1	0	-5.043744	-0.645309	2.549764
32	1	0	-3.786993	-0.301990	-2.305462
33	1	0	-2.236852	-0.806424	2.851955
34	1	0	0.405156	-1.450913	2.244454
35	1	0	1.496647	-0.628040	-1.726147
36	1	0	-0.897014	0.200449	-2.191256
37	1	0	-0.661098	-1.520171	-1.995563
38	1	0	1.234044	0.629316	2.860050
39	1	0	-0.120428	1.356239	1.986382
40	1	0	2.756521	-1.799493	1.436569
41	1	0	3.140356	-1.447561	-0.263299
42	1	0	4.298867	1.754412	1.649427
43	1	0	2.018193	2.261573	1.111459
44	1	0	5.159447	-1.233433	1.380090
45	1	0	6.175187	0.145692	0.940197
46	1	0	5.736944	-0.110773	2.624056
47	1	0	2.656311	2.637222	-1.279677
48	1	0	1.693424	1.896794	-2.581668
49	1	0	3.779306	0.972376	-2.581823
50	1	0	1.087680	-3.203023	0.902163
51	1	0	-0.289303	-2.747893	-0.142298
52	1	0	-1.292912	4.511260	1.059694
53	1	0	-2.217996	3.498159	-0.103036
54	1	0	-0.994881	4.647264	-0.707610

Rotational constants (GHZ): 0.2720507 0.1368033 0.117488

Leave Link 202 at Wed Dec 21 16:54:30 2022, MaxMem= 6442450944 cpu:

(Enter /public1/home/sc81486/software/g09/l301.exe)

Conformer **2b**

Center Number	Atomic Number	Atomic Type	Coordinates (Angstroms) X	Y	Z
1	6	0	5.391851	0.033806	0.847218
2	6	0	5.687921	-0.516924	-0.417503
3	6	0	4.683333	-0.961275	-1.268142
4	6	0	3.364913	-0.840793	-0.818145
5	6	0	3.043908	-0.291999	0.452711
6	6	0	4.082460	0.149703	1.291468
7	7	0	2.174316	-1.194507	-1.437666
8	6	0	1.128176	-0.892908	-0.590084
9	6	0	1.609059	-0.331570	0.567902
10	6	0	-0.313603	-1.049093	-0.962002
11	7	0	-1.119306	-1.126179	0.353293
12	6	0	-0.760859	0.140103	1.216646
13	6	0	0.698639	0.060698	1.690773
14	6	0	-0.859325	0.142757	-1.800989
15	6	0	-3.024817	0.086226	-0.708716
16	6	0	-2.627679	-1.135705	0.082735
17	6	0	-4.252320	0.284744	-1.195449
18	6	0	-1.841683	0.983897	-0.953872
19	6	0	-5.441680	-0.615690	-1.032360
20	6	0	-1.199301	1.426391	0.399553
21	6	0	-2.210754	2.225215	1.283575
22	8	0	-3.164905	1.339822	1.853025
23	6	0	-0.767241	-2.359902	1.157802
24	17	0	-1.174145	-3.891227	0.335608
25	6	0	-0.043231	2.446168	0.201830
26	8	0	0.576301	2.926274	1.127818
27	8	0	0.126337	2.822348	-1.071273
28	6	0	1.135570	3.840116	-1.294640
29	1	0	6.205806	0.371096	1.481600
30	1	0	6.723671	-0.595142	-0.733753
31	1	0	4.915712	-1.385055	-2.240924
32	1	0	3.865621	0.576375	2.266783
33	1	0	2.099900	-1.641689	-2.339441
34	1	0	-0.496416	-1.999685	-1.470356
35	1	0	-1.434575	0.056380	2.070011
36	1	0	0.957868	1.038712	2.102919
37	1	0	0.770154	-0.641346	2.533670
38	1	0	-1.395689	-0.232313	-2.677025
39	1	0	-0.020548	0.744424	-2.150064
40	1	0	-2.843198	-2.069949	-0.438088
41	1	0	-3.103071	-1.161392	1.067501
42	1	0	-4.424721	1.200359	-1.760980
43	1	0	-2.136875	1.872326	-1.515294
44	1	0	-5.212373	-1.542964	-0.497840
45	1	0	-6.239258	-0.102997	-0.479261
46	1	0	-5.862984	-0.884525	-2.008984
47	1	0	-2.695545	2.982344	0.649883
48	1	0	-1.644594	2.741623	2.067454
49	1	0	-3.745251	1.852992	2.436265
50	1	0	0.302929	-2.365168	1.330081
51	1	0	-1.328322	-2.310994	2.090314
52	1	0	1.112851	4.033288	-2.366087
53	1	0	2.113125	3.467345	-0.982908
54	1	0	0.889300	4.740282	-0.728622

Rotational constants (GHZ): 0.2610652 0.1437528 0.1128925

Leave Link 202 at Wed Dec 21 17:08:33 2022, MaxMem= 6442450944 cpu:

(Enter /public1/home/sc81486/software/g09/l301.exe)

Conformer **2c**

Center Number	Atomic Number	Atomic Type	Coordinates (Angstroms) X	Y	Z
1	6	0	5.357830	-0.214450	0.719047
2	6	0	5.677737	-0.596584	-0.600764
3	6	0	4.689418	-0.926309	-1.519811
4	6	0	3.362775	-0.861113	-1.083455
5	6	0	3.016753	-0.476083	0.239986
6	6	0	4.039895	-0.152802	1.149368
7	7	0	2.185155	-1.137517	-1.763671
8	6	0	1.122087	-0.926916	-0.909805
9	6	0	1.579863	-0.519097	0.318845
10	6	0	-0.313225	-1.030718	-1.327115
11	7	0	-1.161013	-1.203811	-0.038624
12	6	0	-0.793334	-0.040428	0.939821
13	6	0	0.639557	-0.243001	1.452017
14	6	0	-0.819926	0.230730	-2.081113
15	6	0	-3.003575	0.159605	-1.028990
16	6	0	-2.655091	-1.155387	-0.373496
17	6	0	-4.218858	0.452748	-1.497590
18	6	0	-1.784575	1.029671	-1.173688
19	6	0	-5.440275	-0.416904	-1.437566
20	6	0	-1.141446	1.327250	0.221187
21	6	0	-2.123293	2.116353	1.142318
22	8	0	-3.146710	1.247215	1.604660
23	6	0	-0.851084	-2.572651	0.534583
24	17	0	-1.671273	-2.908076	2.079632
25	6	0	0.060703	2.286963	-0.009752
26	8	0	0.399966	2.701904	-1.095631
27	8	0	0.624370	2.690017	1.141237
28	6	0	1.728609	3.621550	1.009829
29	1	0	6.159964	0.031619	1.408137
30	1	0	6.719222	-0.635372	-0.905065
31	1	0	4.940243	-1.221979	-2.534417
32	1	0	3.805397	0.136091	2.170385
33	1	0	2.126336	-1.397727	-2.737120
34	1	0	-0.496296	-1.936933	-1.916072
35	1	0	-1.500202	-0.165540	1.758601
36	1	0	0.920487	0.656695	2.003573
37	1	0	0.642287	-1.054296	2.192809
38	1	0	-1.361123	-0.064138	-2.984427
39	1	0	0.033649	0.840700	-2.374426
40	1	0	-2.846277	-2.021664	-1.016467
41	1	0	-3.179873	-1.298668	0.572490
42	1	0	-4.352808	1.427732	-1.965639
43	1	0	-2.029019	1.977444	-1.657434
44	1	0	-5.250793	-1.398532	-0.991881
45	1	0	-6.228885	0.068022	-0.847955
46	1	0	-5.854733	-0.576468	-2.440852
47	1	0	-2.538276	2.952748	0.560742
48	1	0	-1.559991	2.532592	1.984918
49	1	0	-3.712697	1.740127	2.218528
50	1	0	-1.193067	-3.305286	-0.195796
51	1	0	0.221511	-2.652817	0.679908
52	1	0	2.011256	3.873967	2.030810
53	1	0	1.406385	4.508376	0.461612
54	1	0	2.555654	3.142235	0.482099

```
Rotational constants (GHZ):      0.2774849      0.1351331      0.1191052
Leave Link  202 at Wed Dec 21 17:10:43 2022, MaxMem=  6442450944 cpu:
(Enter /public1/home/sc81486/software/g09/l301.exe)
Standard basis: 6-311G(2d,p) (5D, 7F)
```

Conformer **2d**

Center Number	Atomic Number	Atomic Type	Coordinates (Angstroms) X	Y	Z
1	6	0	-5.386629	0.009585	-0.662190
2	6	0	-5.645613	-0.712400	0.521818
3	6	0	-4.615063	-1.247749	1.284267
4	6	0	-3.308939	-1.039669	0.830983
5	6	0	-3.024247	-0.313982	-0.357172
6	6	0	-4.088921	0.212671	-1.110093
7	7	0	-2.100259	-1.456119	1.370111
8	6	0	-1.076738	-1.011428	0.560833
9	6	0	-1.591625	-0.305080	-0.499033
10	6	0	0.375013	-1.191832	0.881467
11	7	0	1.157548	-1.052697	-0.444740
12	6	0	0.755805	0.319725	-1.101506
13	6	0	-0.709390	0.271268	-1.562887
14	6	0	0.914699	-0.128627	1.880240
15	6	0	3.060588	0.036652	0.751074
16	6	0	2.671744	-1.067227	-0.202264
17	6	0	4.294071	0.194426	1.237813
18	6	0	1.862154	0.852228	1.150792
19	6	0	5.501582	-0.639368	0.924187
20	6	0	1.177670	1.478355	-0.107804
21	6	0	2.151992	2.432845	-0.867604
22	8	0	3.094871	1.667294	-1.607104
23	6	0	0.815888	-2.159681	-1.420232
24	17	0	1.271973	-3.787189	-0.846732
25	6	0	0.017515	2.386897	0.392757
26	8	0	-0.271904	2.537346	1.558364
27	8	0	-0.566268	3.072485	-0.605181
28	6	0	-1.619566	3.988152	-0.207875
29	1	0	-6.220107	0.409638	-1.231502
30	1	0	-6.672705	-0.854062	0.843973
31	1	0	-4.818765	-1.804852	2.194130
32	1	0	-3.902290	0.765619	-2.026909
33	1	0	-1.997468	-1.988678	2.221387
34	1	0	0.583528	-2.204946	1.235750
35	1	0	1.415122	0.382960	-1.967641
36	1	0	-0.998698	1.291016	-1.826031
37	1	0	-0.776462	-0.298662	-2.500593
38	1	0	1.477190	-0.618810	2.679695
39	1	0	0.076571	0.404590	2.326837
40	1	0	2.914079	-2.061974	0.175036
41	1	0	3.128022	-0.941605	-1.188440
42	1	0	4.455901	1.020703	1.929823
43	1	0	2.140228	1.656068	1.835271
44	1	0	5.282777	-1.491123	0.272549
45	1	0	6.271050	-0.032582	0.429391
46	1	0	5.955319	-1.027221	1.844571
47	1	0	2.650214	3.071599	-0.124067
48	1	0	1.571427	3.075797	-1.538036
49	1	0	3.705698	2.278303	-2.047210
50	1	0	-0.257361	-2.165475	-1.573205
51	1	0	1.356680	-1.957658	-2.344145
52	1	0	-1.932016	4.479104	-1.128331
53	1	0	-1.232277	4.712422	0.510710
54	1	0	-2.445742	3.432192	0.239780

Rotational constants (GHZ): 0.2620693 0.1443811 0.111844

Leave Link 202 at Wed Dec 21 17:07:39 2022, MaxMem= 6442450944 cpu:

(Enter /public1/home/sc81486/software/g09/l301.exe)

2.3. Experimental and calculated ECD spectra of 1 and 2

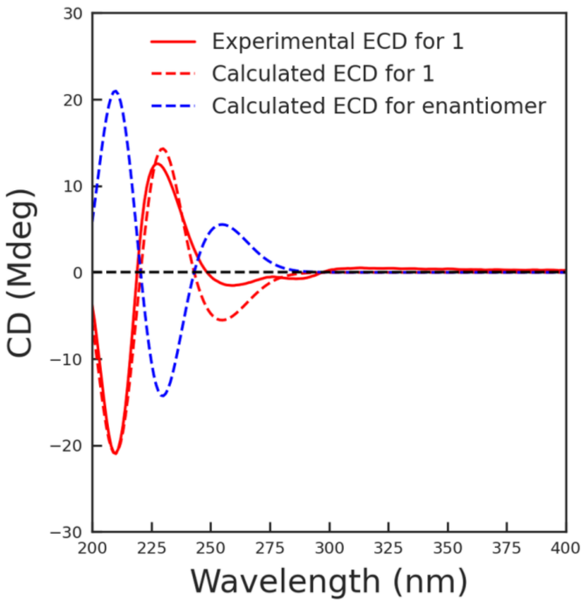

Figure S4. Calculated ECDs and experimental of **1.**

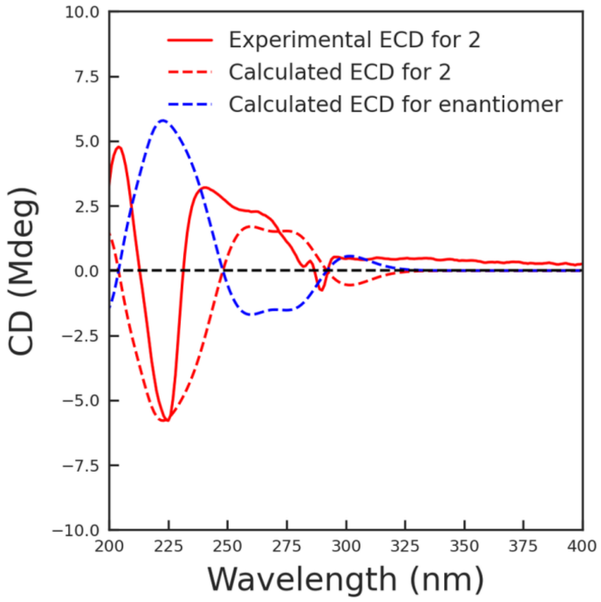

Figure S5. Calculated ECDs and experimental of **2.**

3. Supplementary figures

Figure S6. [1] **H NMR spectrum of 1 (CD_3OD, 400 MHz)**

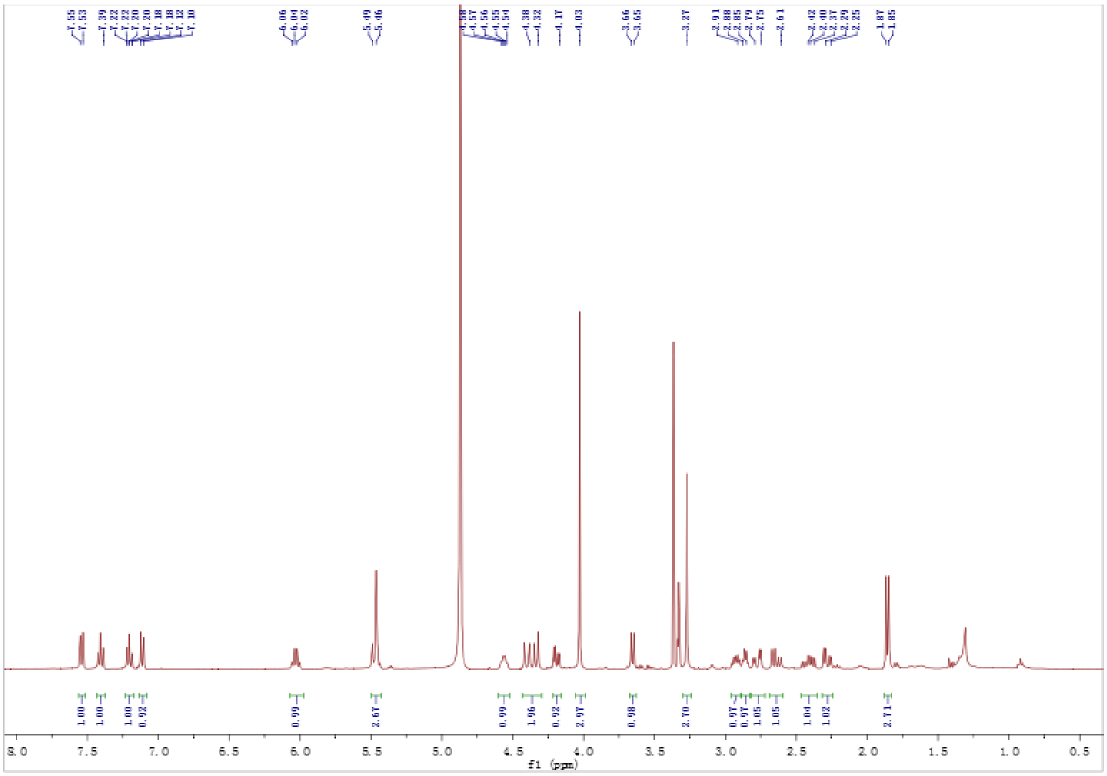

Figure S7. ^{13}C NMR spectrum of 1 (CD_3OD, 100 MHz)

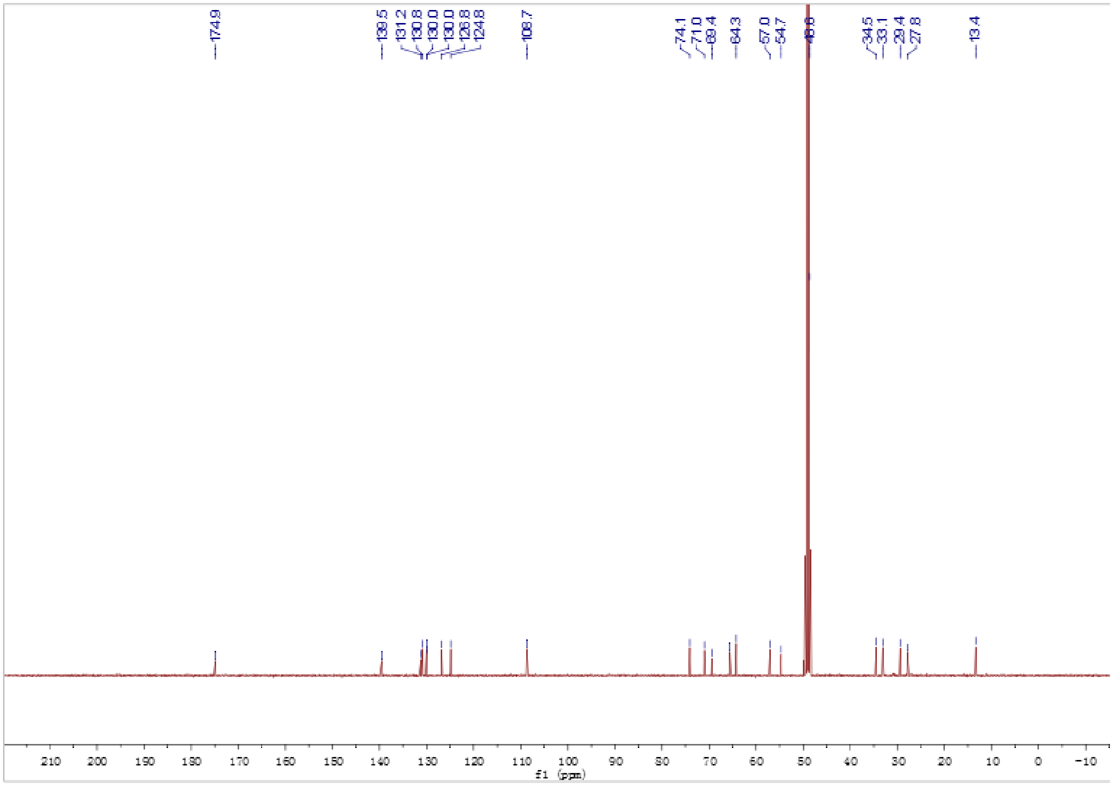

Figure S8. HSQC spectrum of 1 (CD_3OD, 400 MHz).

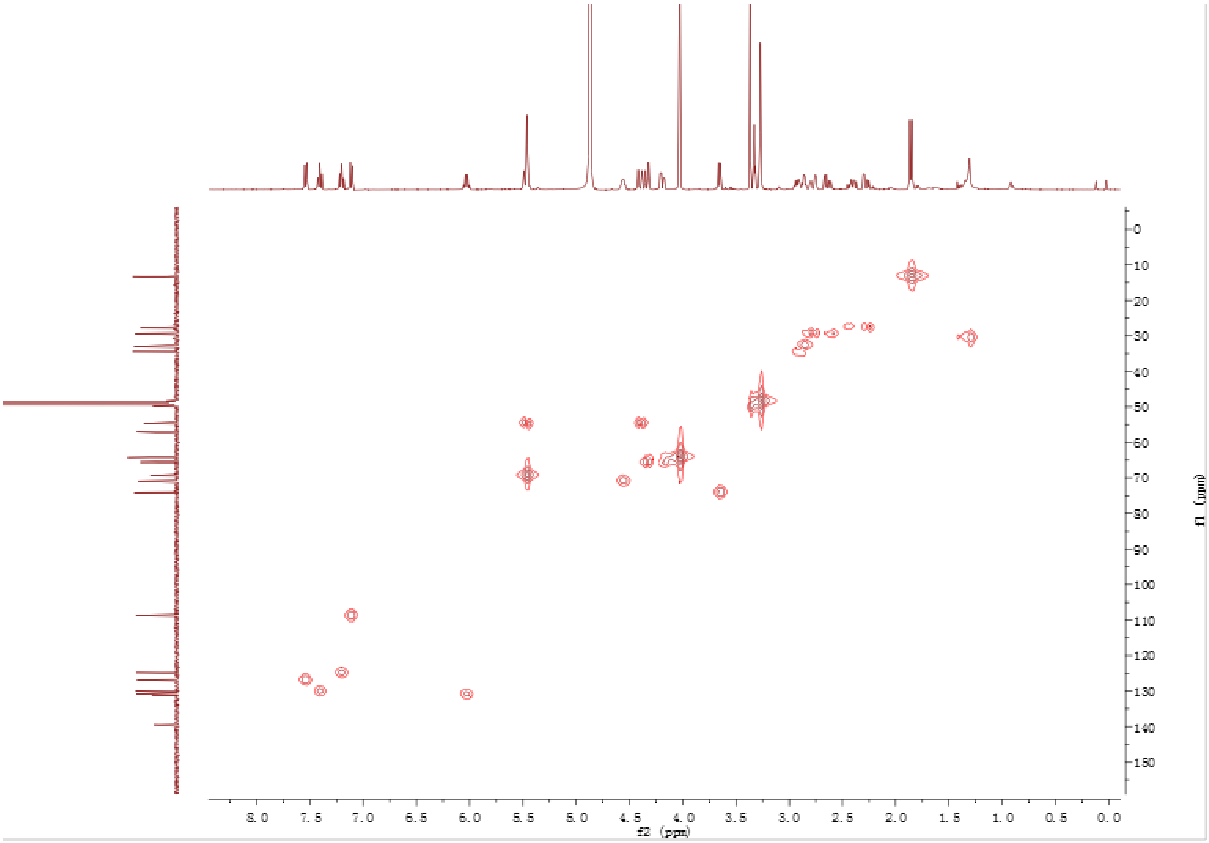

Figure S9. HMBC spectrum of 1 (CD_3OD, 400 MHz).

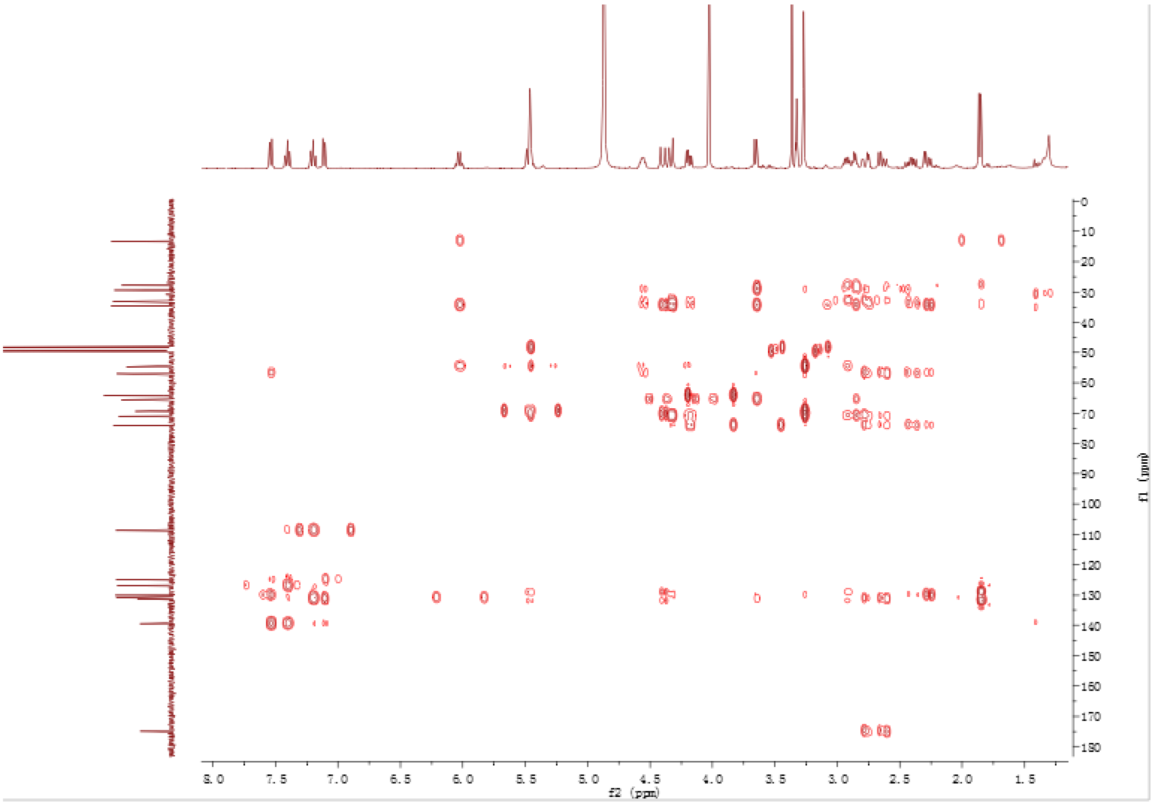

Figure S10. ROESY spectrum of 1 (CD_3OD, 400 MHz).

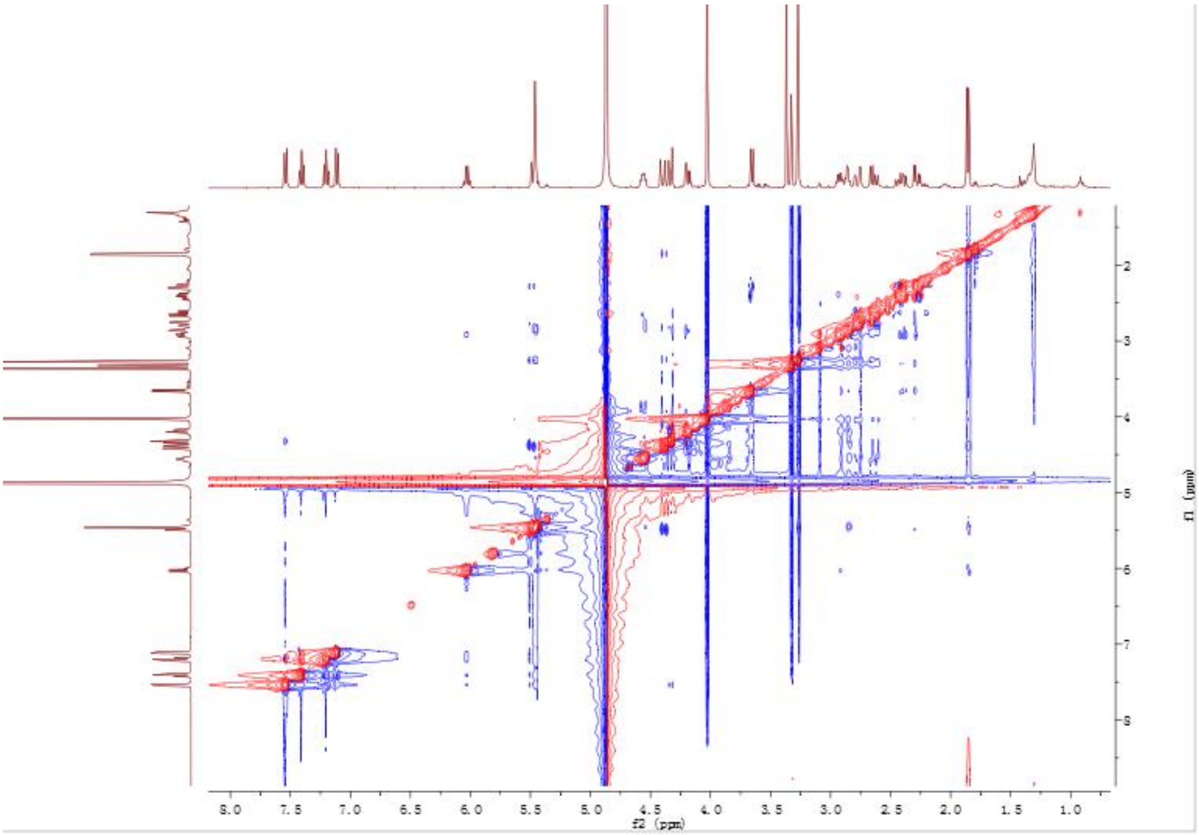

Figure S11. ^{1}H-^{1}H **COSY spectrum of 1 (CD_3OD, 400 MHz).**

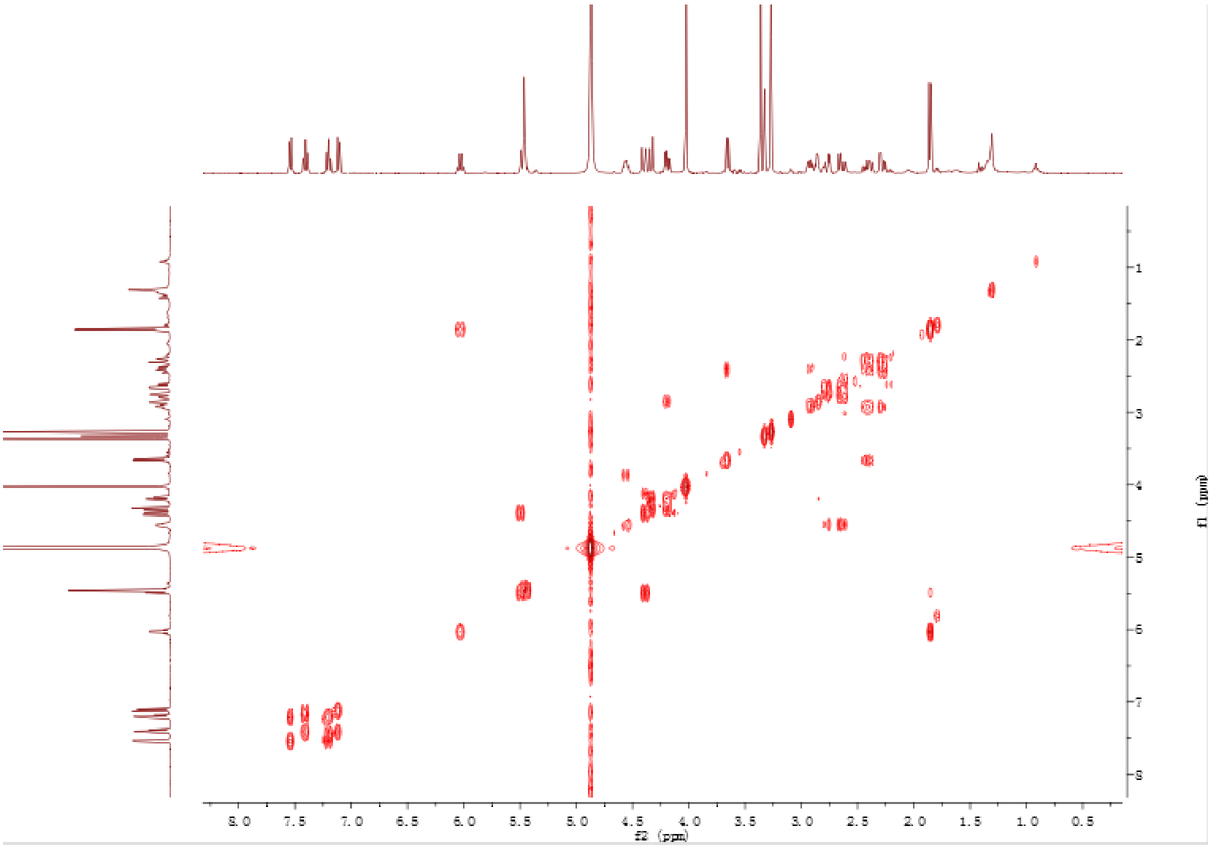

Figure S12. HR-ESI-MS spectrum of 1

Formula Predictor Report - GL-71.lcd

Data File: E:\DATA\2022\1125\GL-71.lcd

Elmt	Val.	Min	Max	Elmt	Val.	Min	Max	Elmt	Val.	Min	Max	Elmt	Val.	Min	Max	Use Adduct
H	1	5	100	F	1	0	0	Cl	1	0	5	Ag	1	0	0	None
2H	1	0	0	Na	1	0	0	Co	2	0	0	I	3	0	0	
B	3	0	0	Mg	2	0	0	Cu	2	0	0	Ir	3	0	0	
C	4	5	60	Si	4	0	0	Se	2	0	0					
N	3	0	5	P	3	0	0	Br	1	0	0					
O	2	0	30	S	2	0	0	Pd	2	0	0					

Error Margin (ppm):	5	DBE Range:	not fixed	Electron Ions:	both
HC Ratio:	unlimited	Apply N Rule:	no	Use MSn Info:	yes
Max Isotopes:	all	Isotope RI (%):	1.00	Isotope Res:	10000
MSn Iso RI (%):	75.00	MSn Logic Mode:	OR	Max Results:	30

Event#: 1 MS(E+) Ret. Time : 0.453 -> 0.813 Scan# : 69 -> 123

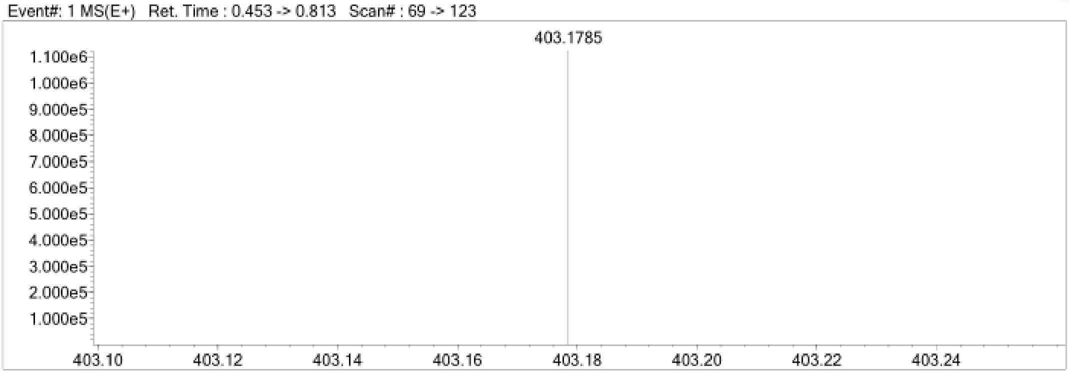

Measured region for 403.1785 m/z

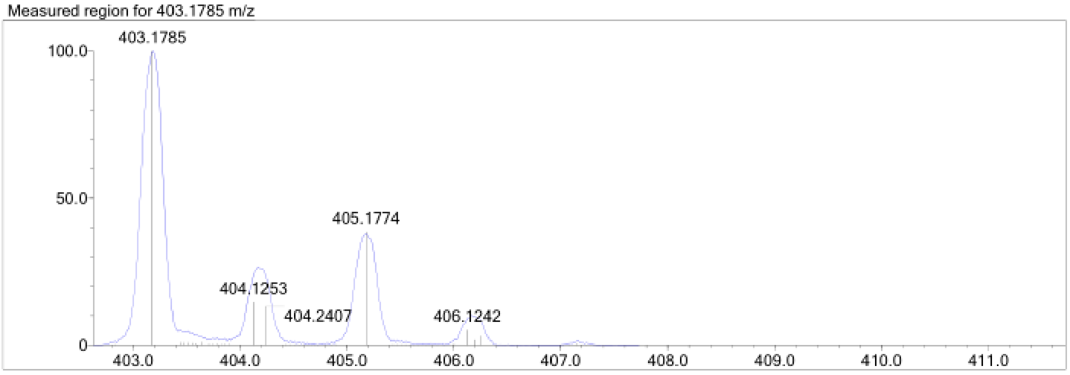

C22 H28 N2 O3 Cl M+ : Predicted region for 403.1783 m/z

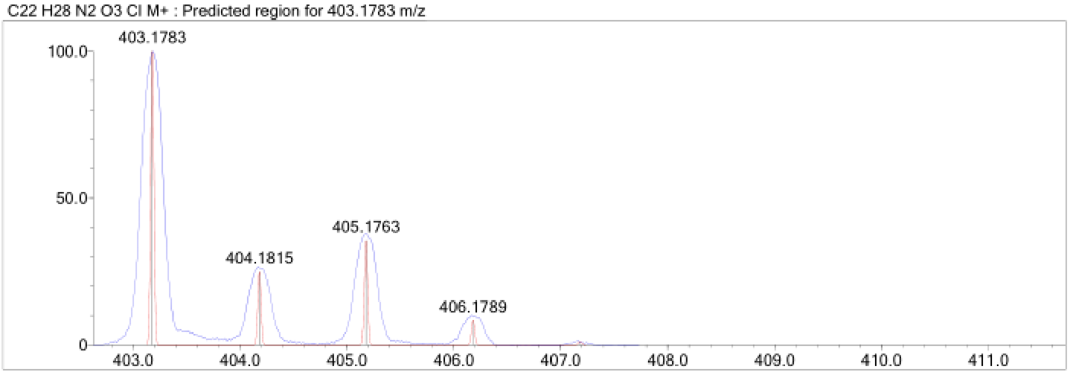

Formula (M)	Ion	Meas. m/z	Pred. m/z	Df. (mDa)	Df. (ppm)	DBE
C22 H28 N2 O3 Cl	M+	403.1785	403.1783	0.2	0.50	9.5

Figure S13. ORD spectrum of 1

Rudolph Research Analytical

This sample was measured on an Autopol VI, Serial #91058
Manufactured by Rudolph Research Analytical, Hackettstown, NJ, USA.

Measurement Date : Thursday, 08-DEC-2022

Set Temperature : OFF

Time Delay : Disabled

Delay between Measurement : Disabled

n	Average	Std.Dev.	% RSD	Maximum	Minimum
5	-102.07	0.58	-0.56	-101.50	-103.00

S.No	Sample ID	Time	Result	Scale	OR °Arc	WLG.nm	Lg.mm	Conc.g/100ml	Temp.
1	GL-71	08:26:55 PM	-102.17	SR	-0.0613	589	100.00	0.060	18.5
2	GL-71	08:27:03 PM	-103.00	SR	-0.0618	589	100.00	0.060	18.5
3	GL-71	08:27:12 PM	-102.00	SR	-0.0612	589	100.00	0.060	18.4
4	GL-71	08:27:20 PM	-101.50	SR	-0.0609	589	100.00	0.060	18.4
5	GL-71	08:27:28 PM	-101.67	SR	-0.0610	589	100.00	0.060	18.4

Figure S14. UV spectrum of 1

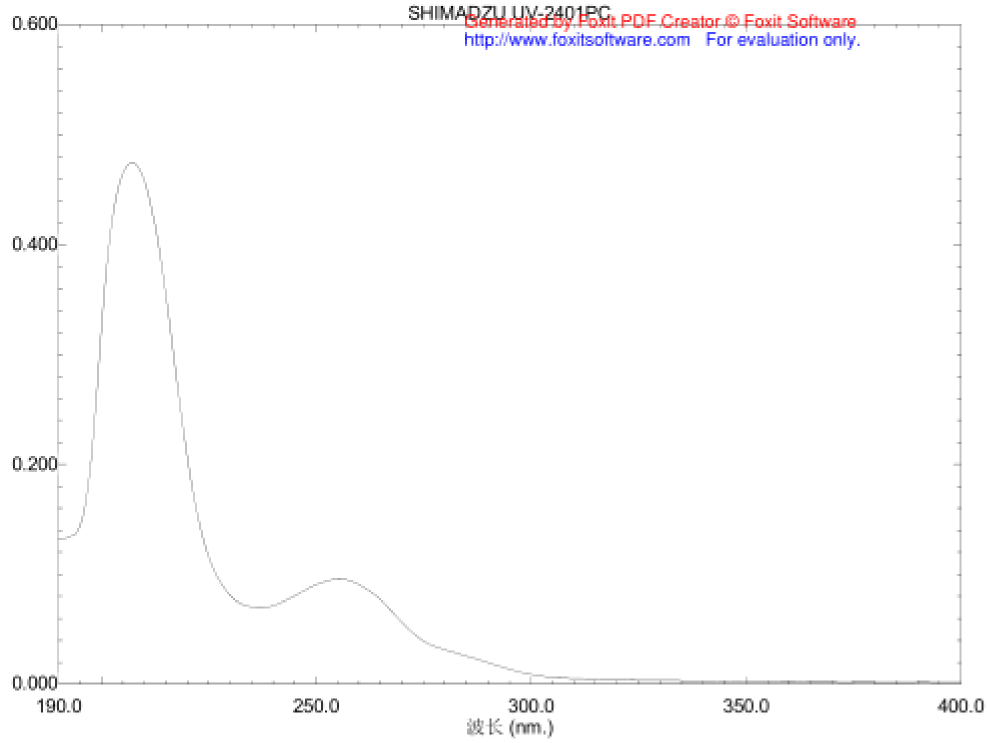

文件名: GL-71

GL-71 ———

样品浓度：0.0063毫克/毫升
溶剂：甲醇

创建于: 12:24 22-12-12
数据: 原始

测量模式: Abs.
扫描速度: 中速
狭缝: 5.0
采样间隔: 0.5

峰.	波长 (nm.)	Abs.
1	207.00	0.4742
2	254.50	0.0949
3	279.50	0.0320

Figure S15. IR spectrum of 1

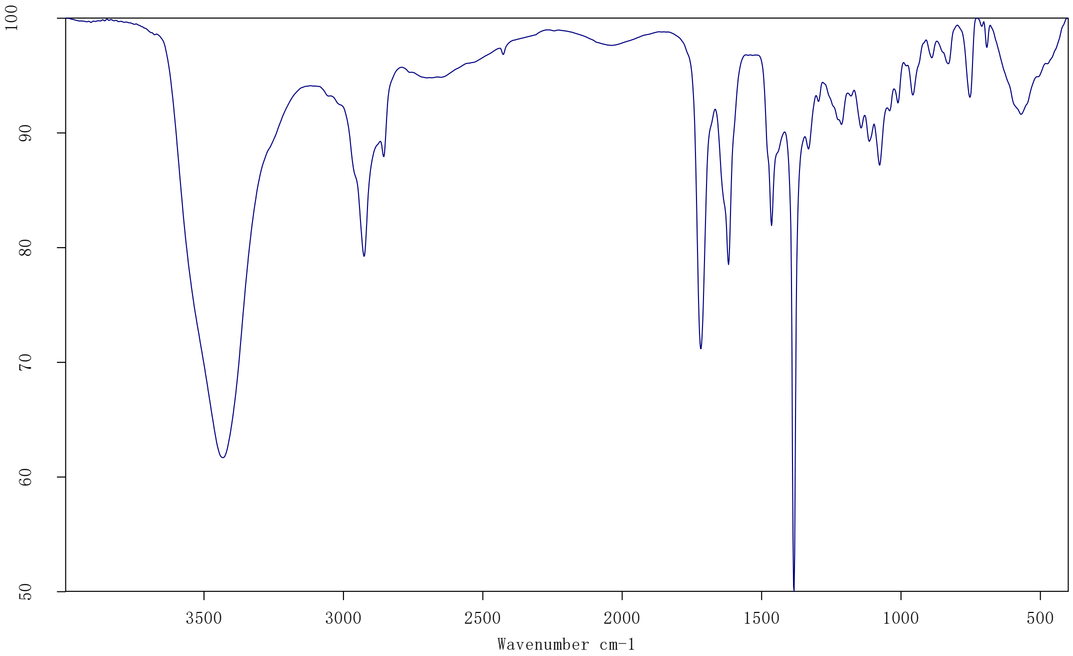

Sample Name: GL-71
Sample Form: KBr
Path of File: E:\data
Date of Measurement: 2022/11/29
Resolution: 4
Aperture Setting: 6 mm
Number of Background Scans: 16
Number of Sample Scans: 16
Beamsplitter Setting: KBr
Source Setting: MIR
Instrument Type: BRUKER VERTEX 70
Soft Version: OPUS8.1

Figure S16. ECD spectrum of 1

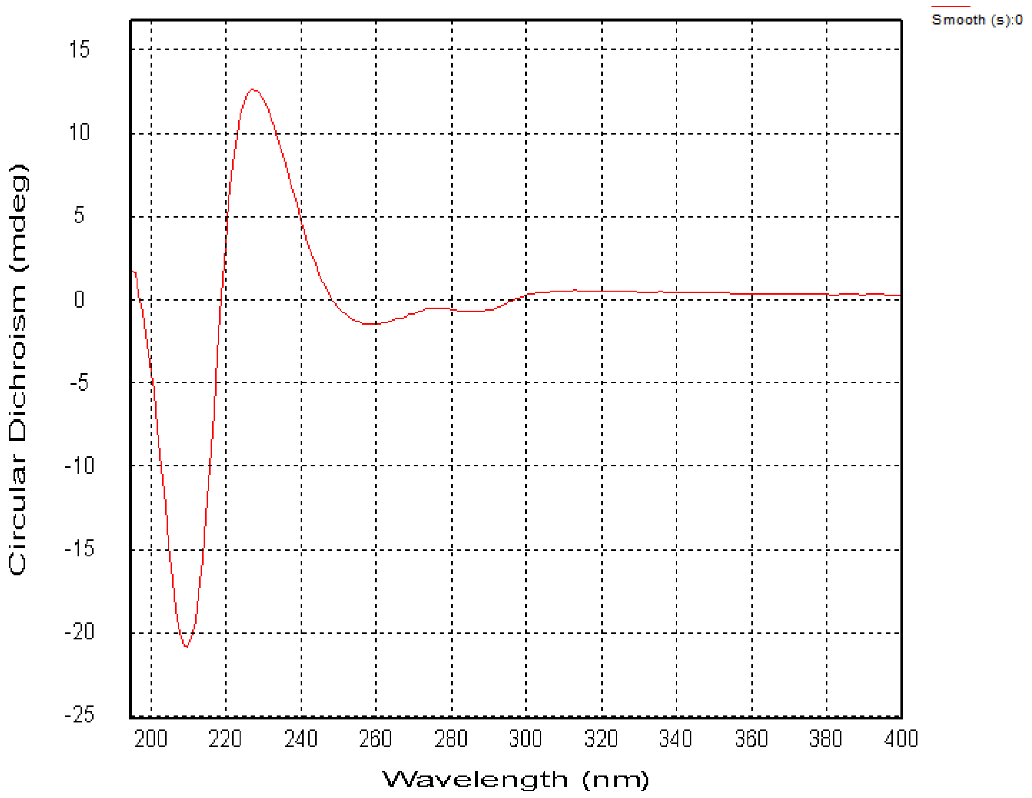

Figure S17. ^{1}H NMR spectrum of 2 (CD_3OD, 400 MHz)

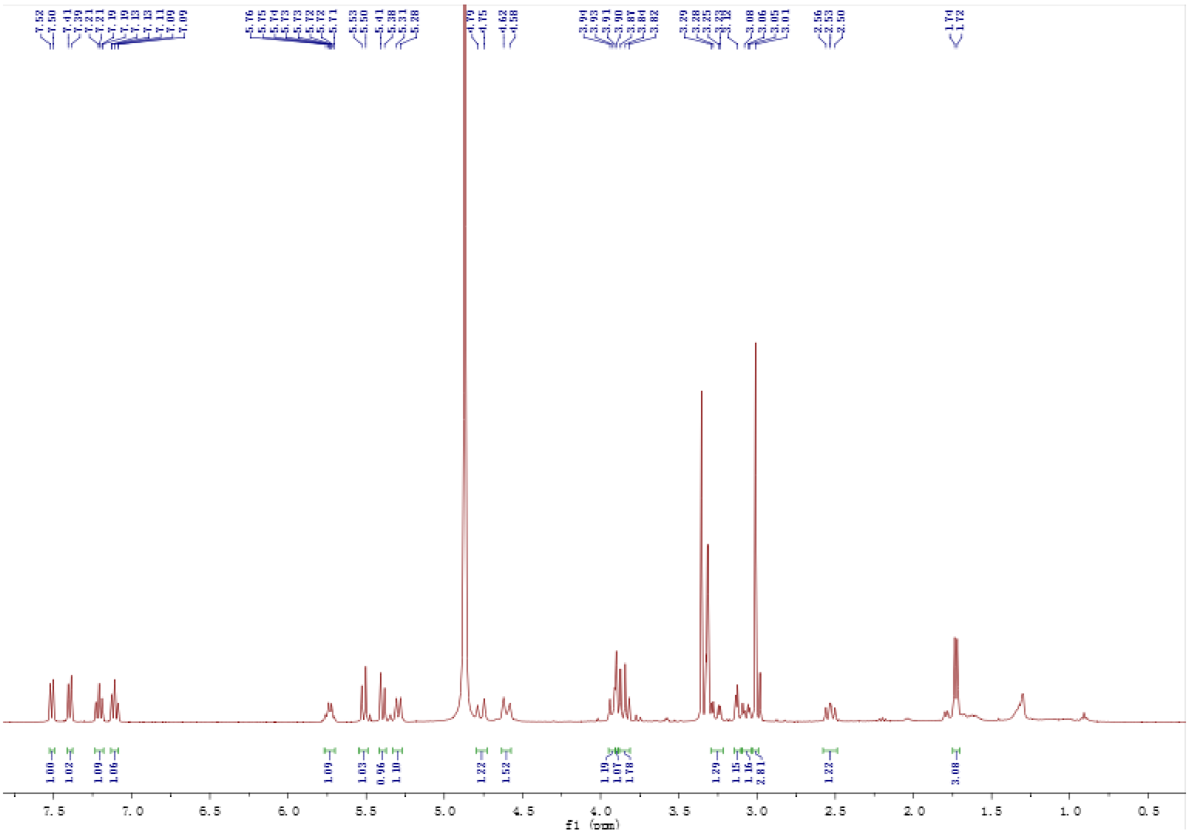

Figure S18. ^{13}C **NMR spectrum of 2 (**CD_3OD**, 100 MHz)**

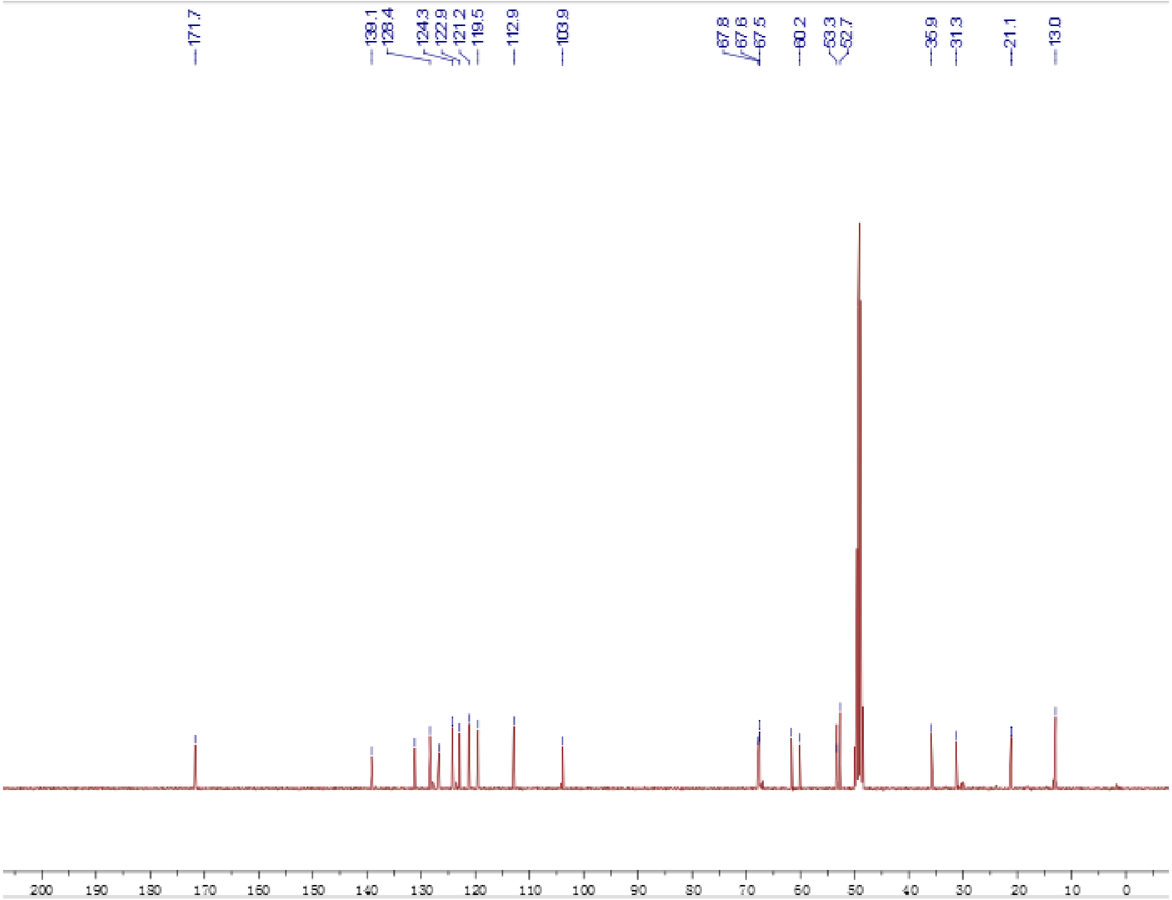

Figure S19. HSQC spectrum of 2 (CD₃OD, 400 MHz).

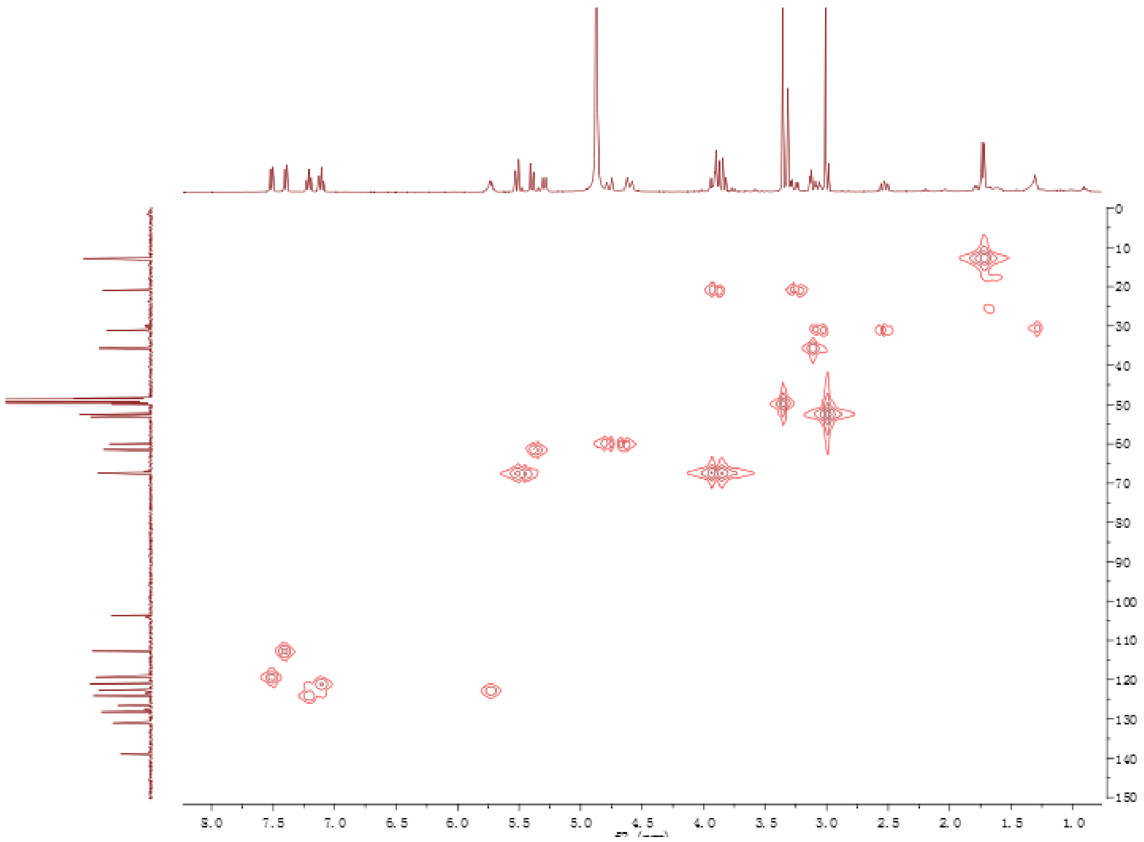

Figure S20. HMBC spectrum of 2 (CD_3OD, 400 MHz).

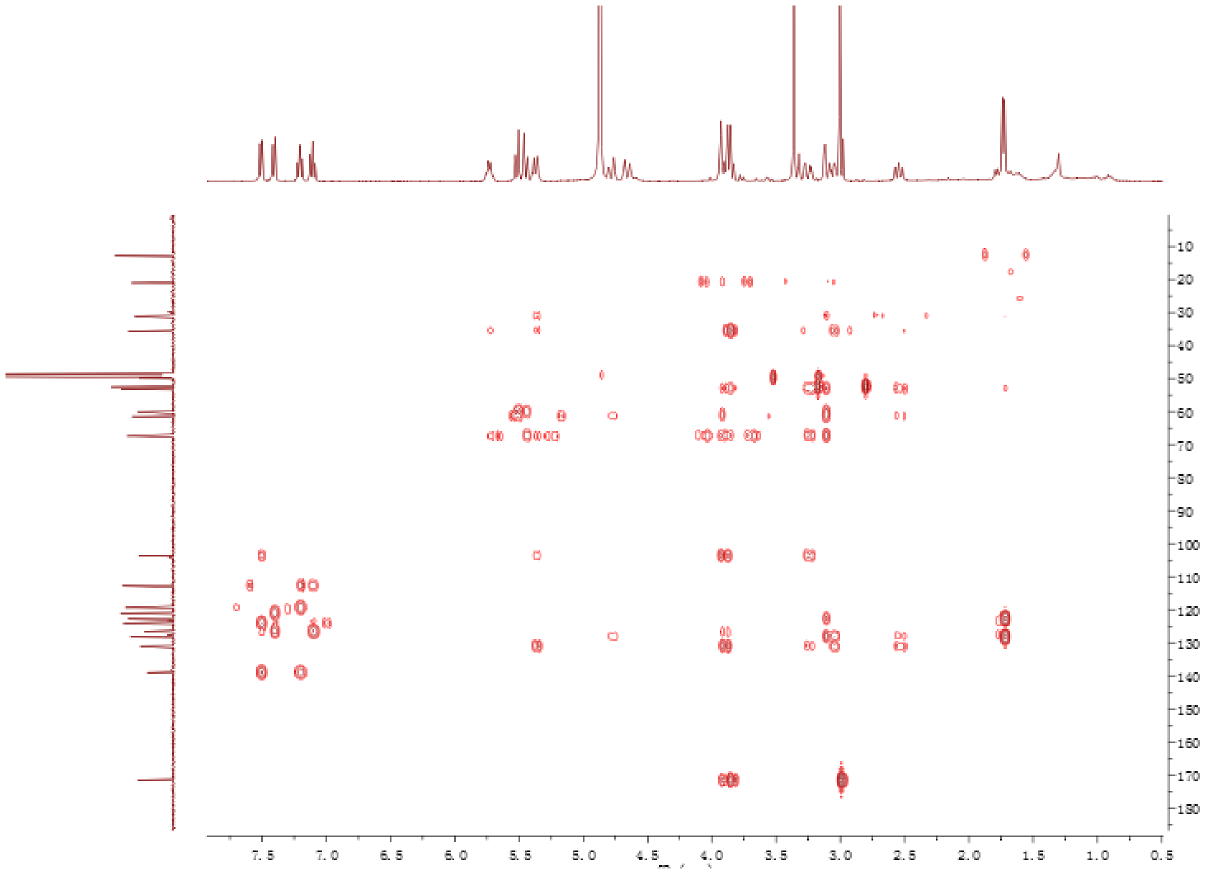

Figure S21. ROESY spectrum of 2 (CD_3OD, 400 MHz).

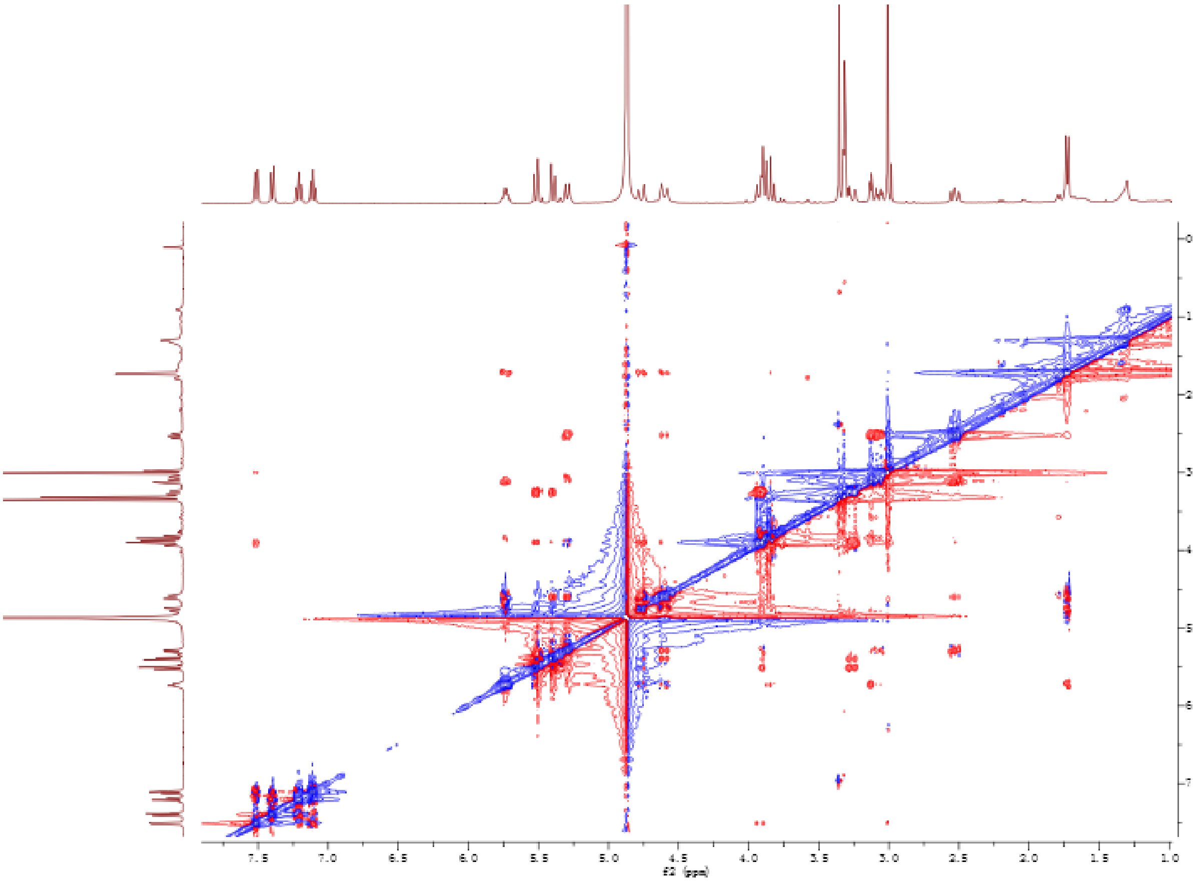

Figure S22. ^1H-^1H **spectrum of 2 (CD_3OD, 400 MHz).**

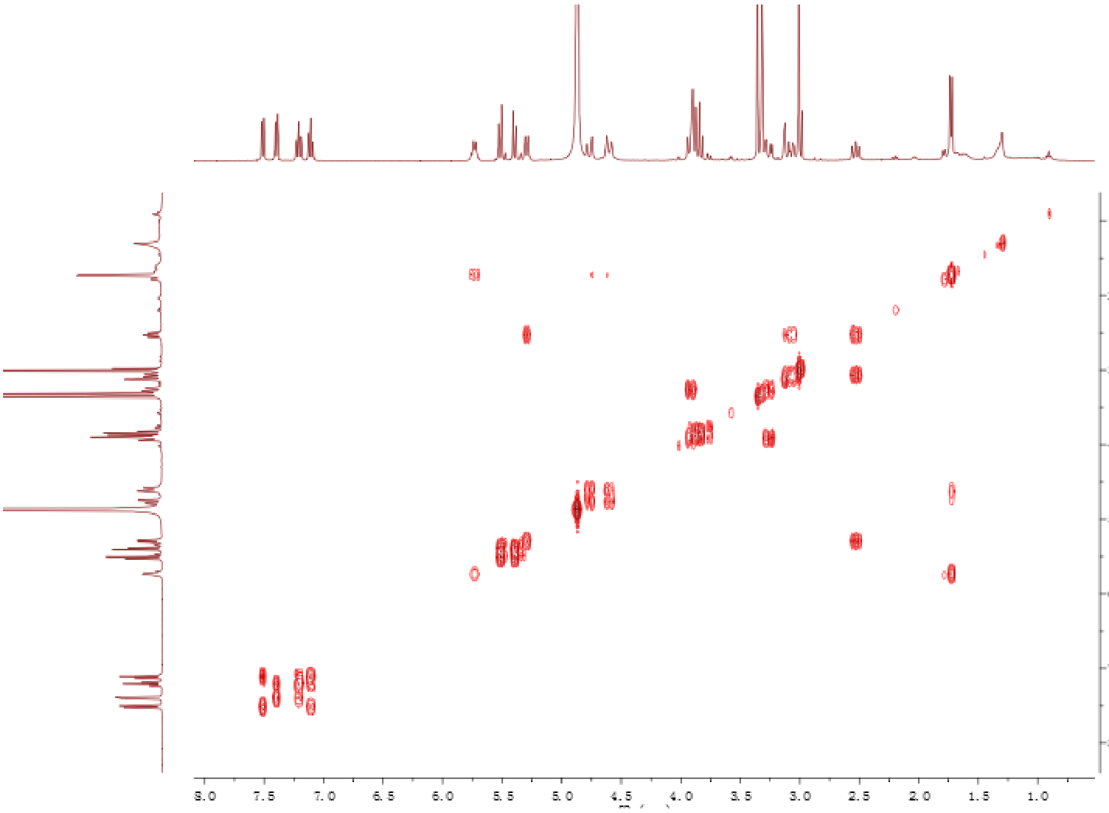

Figure S23. HR-ESI-MS spectrum of 2

Formula Predictor Report - GL-72.lcd

Data File: E:\DATA\2022\1125\1\GL-72.lcd

Elmt	Val.	Min	Max	Elmt	Val.	Min	Max	Elmt	Val.	Min	Max	Elmt	Val.	Min	Max	Use Adduct
H	1	5	100	F	1	0	0	Cl	1	0	5	Ag	1	0	0	None
2H	1	0	0	Na	1	0	0	Co	2	0	0	I	3	0	0	
B	3	0	0	Mg	2	0	0	Cu	2	0	0	Ir	3	0	0	
C	4	5	60	Si	4	0	0	Se	2	0	0					
N	3	0	5	P	3	0	0	Br	1	0	0					
O	2	0	30	S	2	0	0	Pd	2	0	0					

Error Margin (ppm):	5	DBE Range:	not fixed	Electron Ions:	both
HC Ratio:	unlimited	Apply N Rule:	no	Use MSn Info:	yes
Max Isotopes:	all	Isotope RI (%):	1.00	Isotope Res:	10000
MSn Iso RI (%):	75.00	MSn Logic Mode:	OR	Max Results:	30

Event#: 1 MS(E+) Ret. Time : 0.613 Scan# : 93

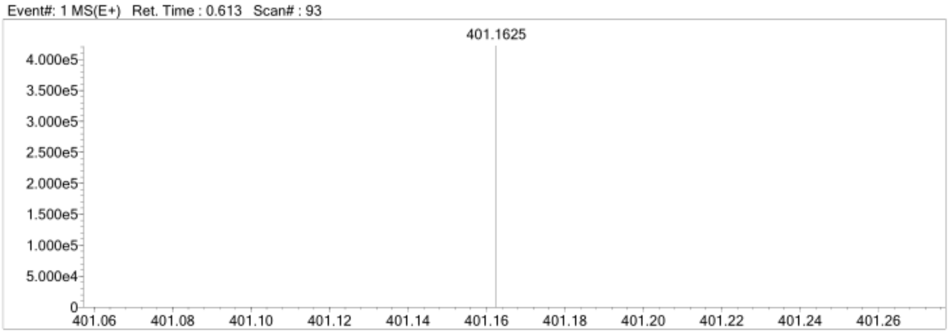

Measured region for 401.1625 m/z

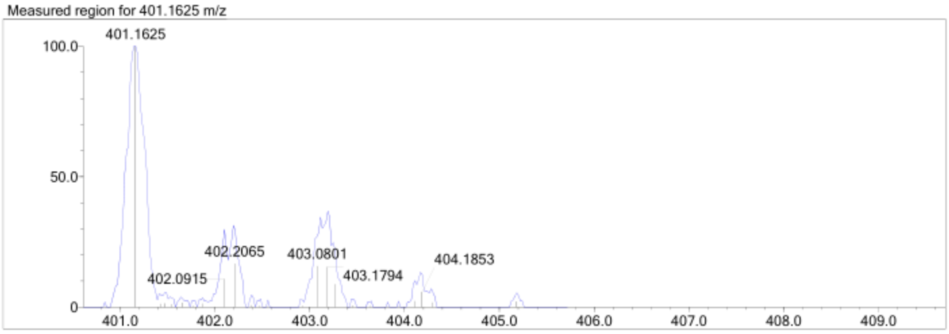

C22 H26 N2 O3 Cl M+ : Predicted region for 401.1626 m/z

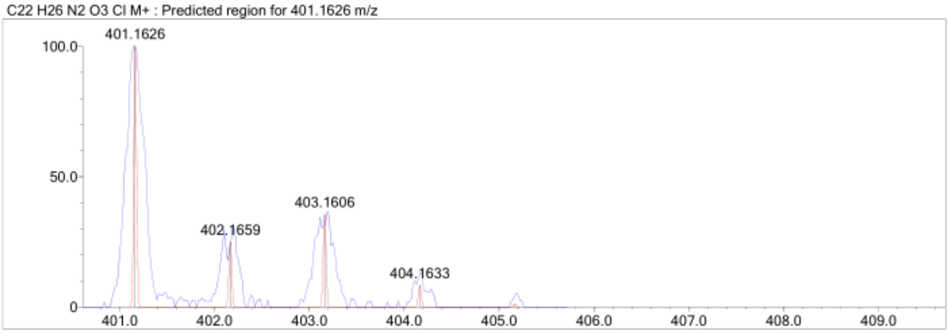

Formula (M)	Ion	Meas. m/z	Pred. m/z	Df. (mDa)	Df. (ppm)	DBE
C22 H26 N2 O3 Cl	M+	401.1625	401.1626	-0.1	-0.25	10.5

Figure S24. ORD spectrum of 2

Rudolph Research Analytical

This sample was measured on an Autopol VI, Serial #91058
Manufactured by Rudolph Research Analytical, Hackettstown, NJ, USA.

Measurement Date : Thursday, 08-DEC-2022

Set Temperature : OFF

Time Delay : Disabled

Delay between Measurement : Disabled

n	Average	Std.Dev.	% RSD	Maximum	Minimum
5	-18.06	1.05	-5.81	-16.57	-19.25

S.No	Sample ID	Time	Result	Scale	OR °Arc	WLG.nm	Lg.mm	Conc.g/100ml	Temp.
1	GL-72	08:21:07 PM	-19.25	SR	-0.0129	589	100.00	0.067	18.5
2	GL-72	08:21:15 PM	-18.06	SR	-0.0121	589	100.00	0.067	18.5
3	GL-72	08:21:24 PM	-16.57	SR	-0.0111	589	100.00	0.067	18.5
4	GL-72	08:21:32 PM	-17.61	SR	-0.0118	589	100.00	0.067	18.5
5	GL-72	08:21:40 PM	-18.81	SR	-0.0126	589	100.00	0.067	18.5

Figure S25. UV spectrum of 2

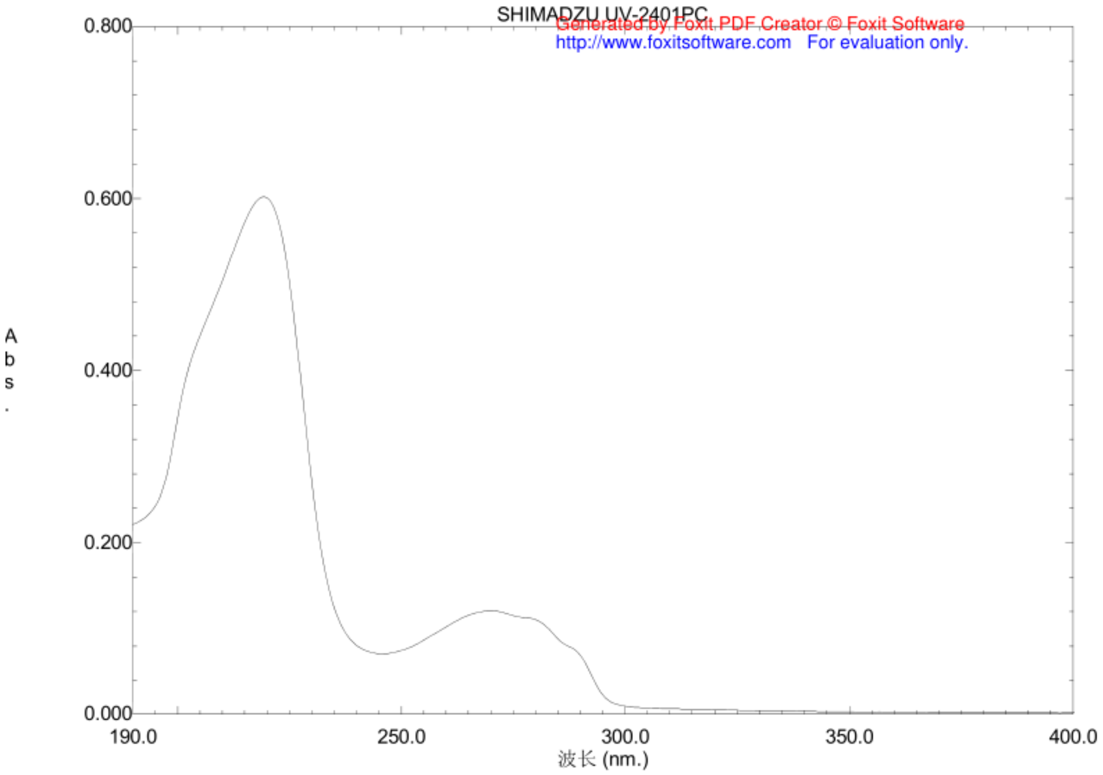

Generated by Foxit PDF Creator © Foxit Software
http://www.foxitsoftware.com For evaluation only.

文件名: GL-72

GL-72 ———

样品浓度：0.0078毫克/毫升
溶剂：甲醇

创建于: 12:30 22-12-12
数据: 原始

测量模式: Abs.
扫描速度: 中速
狭缝: 5.0
采样间隔: 0.5

否.	波长 (nm.)	Abs.
1	219.00	0.6018
2	269.00	0.1199
3	278.50	0.1116
4	285.00	0.0875

Figure S26. IR spectrum of 2

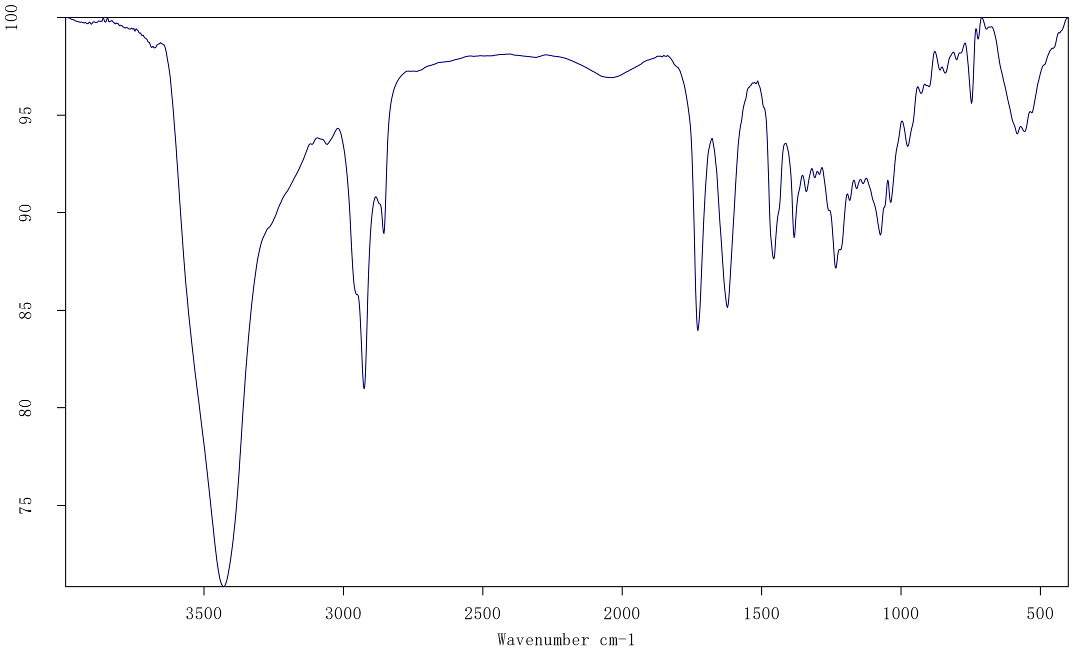

Sample Name: GL-72
Sample Form: KBr
Path of File: E:\data
Date of Measurement: 2022/11/29

Resolution: 4
Aperture Setting: 6 mm
Number of Background Scans: 16
Number of Sample Scans: 16

Beamsplitter Setting: KBr
Source Setting: MIR
Instrument Type: BRUKER VERTEX 70
Soft Version: OPUS8.1

Figure S27. ECD spectrum of 2

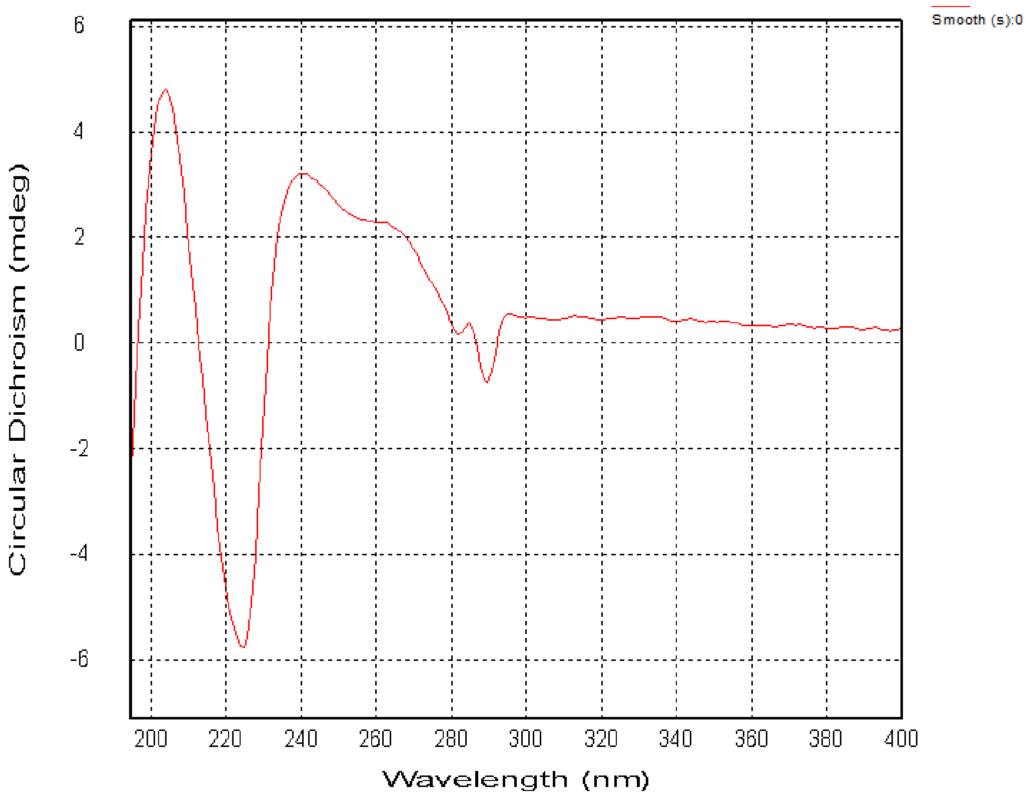

References

(1) Noel M OBoyle, Tim V, ermeersch, Christopher J Flynn, Anita R Maguire Maguire, and Geoffey R Hutchison. Confab - systematic generation of diverse low-energy conformers. Journal of Cheminformatics, 3:3–8, March 2011.

(2) M. J. Frisch, G. W. Trucks, H. B. Schlegel, G. E. Scuseria, M. A. Robb, J. R. Cheeseman, G. Scalmani, V. Barone, B. Mennucci, G. A. Petersson, H. Nakatsuji, M. Caricato, X. Li, H. P. Hratchian, A. F. Izmaylov, J. Bloino, G. Zheng, J. L. Sonnenberg, M. Hada, M. Ehara, K. Toyota, R. Fukuda, J. Hasegawa, M. Ishida, T. Nakajima, Y. Honda, O. Kitao, H. Nakai, T. Vreven, J. A. Montgomery, Jr., J. E. Peralta, F. Ogliaro, M. Bearpark, J. J. Heyd, E. Brothers, K. N. Kudin, V. N. Staroverov, R. Kobayashi, J. Normand, K. Raghavachari, A. Rendell, J. C. Burant, S. S. Iyengar, J. Tomasi, M. Cossi, N. Rega, J. M. Millam, M. Klene, J. E. Knox, J. B. Cross, V. Bakken, C. Adamo, J. Jaramillo, R. Gomperts, R. E. Stratmann, O. Yazyev, A. J. Austin, R. Cammi, C. Pomelli, J. W. Ochterski, R. L. Martin, K. Morokuma, V. G. Zakrzewski, G. A. Voth, P. Salvador, J. J. Dannenberg, S. Dapprich, A. D. Daniels, O. Farkas, J. B. Foresman, J. V. Ortiz, J. Cioslowski, and D. J. Fox. Gaussian 09 Revision D.01. Gaussian Inc. Wallingford CT 2009.
